# Sex Differences in the Alcohol-Mediated Modulation of BLA Network States

**DOI:** 10.1523/ENEURO.0010-22.2022

**Published:** 2022-07-07

**Authors:** Alyssa DiLeo, Pantelis Antonoudiou, Spencer Ha, Jamie L. Maguire

**Affiliations:** 1 Program in Neuroscience, Graduate School of Biomedical Sciences, Tufts University, Boston, Massachusetts 02111; 2 Department of Neuroscience, Tufts School of Medicine, Tufts University, Boston, Massachusetts 02111

**Keywords:** alcohol, basolateral amygdala, GABA, network states, oscillations, sex differences

## Abstract

Alcohol use, reported by 85% of adults in the United States, is highly comorbid with mood disorders, like generalized anxiety disorder and major depression. The basolateral amygdala (BLA) is an area of the brain that is heavily implicated in both mood disorders and alcohol use disorder. Importantly, the modulation of BLA network/oscillatory states via parvalbumin (PV)-positive GABAergic interneurons has been shown to control the behavioral expression of fear and anxiety. Further, PV interneurons express a high density of δ subunit-containing GABA_A_ receptors (GABA_A_Rs), which are sensitive to low concentrations of alcohol. Therefore, we hypothesized that the effects of alcohol may modulate BLA network states that have been associated with fear and anxiety behaviors via δ-GABA_A_Rs on PV interneurons in the BLA. Given the impact of ovarian hormones on the expression of δ-GABA_A_Rs, we also examined the ability of alcohol to modulate local field potentials in the BLA from male and female C57BL/6J and *Gabrd*^−/−^ mice after acute and repeated exposure to alcohol. Here, we demonstrate that acute and repeated alcohol can differentially modulate oscillatory states in male and female C57BL/6J mice, a process that involves δ-GABA_A_Rs. This is the first study to demonstrate that alcohol is capable of altering network states implicated in both anxiety and alcohol use disorders.

## Significance Statement

Alcohol use disorder and mood disorders are highly comorbid. The basolateral amygdala (BLA) is implicated in both disorders, but the mechanisms contributing to their shared pathophysiology remain uncertain. Here we demonstrate that acute and repeated alcohol exposure can alter network oscillations in the BLA that control the behavioral expression of fear and anxiety. These data suggest that alcohol may directly influence network states associated with mood. Further, we demonstrate sex differences in the ability of alcohol to modulate BLA network states, an effect involving δ-GABA_A_ receptors, which may contribute to sex differences in alcohol intake and comorbid mood disorders. These data potentially point to a novel mechanism mediating the effects of alcohol on affective states.

## Introduction

Alcohol is the most widely used drug in the United States, with ∼85% of adults reporting alcohol use in their lifetime. Despite this high rate of use, only ∼5% will go on to develop an alcohol use disorder while most adults continue to drink without reaching this diagnostic criterion (Substance Abuse and Mental Health Services Administration, 2020). The transition from first drink to alcohol dependence is encouraged by both the positive and negative reinforcing effects of alcohol, each with corresponding neurobiological frameworks (Gilpin and Koob, 2008). Comorbid mood disorders, such as major depression and anxiety disorders, contribute to the reinforcing effects of alcohol by pushing individuals to drink to relieve tension in high-stress or high-anxiety situations (Kushner et al., 2011). The basolateral amygdala (BLA) has been identified as a brain region contributing to both alcohol use disorder and anxiety disorders (Silberman et al., 2009; Tye et al., 2011; Agoglia and Herman, 2018).

Accumulating evidence demonstrates a critical role for oscillatory states in the BLA in modulating fear and anxiety-like behaviors (Likhtik et al., 2014; Stujenske et al., 2014; Davis et al., 2017; Antonoudiou et al., 2021). However, the impact of alcohol on these network states has not been explored. Network oscillations within and between brain areas represent a mechanism for the transition between brain and behavioral states. Specifically, particular oscillation frequencies within and between the BLA and medial prefrontal cortex (mPFC) are associated with either a fear (3–6 Hz) or safety (6–12 Hz) state (Davis et al., 2017). This circuit, along with other regions like the hippocampus, has also been shown to contribute to high- and low-anxiety states in mice (Likhtik et al., 2014).

It is well established that the anxiolytic properties of alcohol can motivate consumption and contribute to the high comorbidity of alcohol use disorders and mood disorders (Thomas et al., 2003; Smith and Randall, 2012; Mason et al., 2019). However, it is unclear how alcohol impacts network states underlying modulation of anxiety states. Here we examine the ability of acute, low-dose alcohol to modulate BLA network activity in alcohol-naive mice, using local field potentials (LFPs) to measure network oscillations in the BLA in male and female C57BL/6J mice during acute and repeated exposures to alcohol.

The generation of oscillations is thought to involve the ability of GABAergic interneurons, particularly parvalbumin (PV)-expressing interneurons, to synchronize populations of principal neurons (Bartos et al., 2007; Sohal et al., 2009; Fuchs et al., 2017). Somatic-targeting, fast-spiking PV interneurons exert powerful control over a large network of excitatory principal cells, and, as such, are capable of generating and synchronizing oscillations to orchestrate network communication (McDonald, 1992; Bocchio and Capogna, 2014). There is a critical role for PV interneurons in oscillation generation within the BLA both *ex vivo* and *in vivo*, where PV interneurons can shift oscillatory frequencies and drive behavioral states (Davis et al., 2017; Ozawa et al., 2020; Antonoudiou et al., 2021).

PV interneurons in the BLA express a high density of extrasynaptic δ subunit-containing GABA_A_ receptors (GABA_A_Rs), which are uniquely sensitive to alcohol and play a role in regulating both alcohol consumption and anxiety-like behaviors, including anxiety associated with alcohol withdrawal (Glykys et al., 2007; Melón et al., 2019; Antonoudiou et al., 2021). Tonic inhibition mediated by δ-GABA_A_Rs has been shown to control hippocampal oscillations (Mann and Mody, 2010; Pavlov et al., 2014) and loss of the δ subunit in PV interneurons alters gamma oscillations in the CA3 region of the hippocampus (Ferando and Mody, 2013, 2014). Given the evidence that PV interneurons, which modulate oscillations in the BLA, have a high density of δ subunit expression, we further hypothesized that alcohol acts via δ-GABA_A_Rs in the BLA to modulate oscillations associated with the network communication of fear and anxiety. To test this, we examined the ability of alcohol to alter oscillatory states in the BLA of male and female *Gabrd*^−/−^ mice. Our findings suggest that the ability of alcohol to modulate network states involves δ subunit-containing GABA_A_Rs. We conclude that alcohol can modulate BLA oscillatory states in a sex-specific manner, a process which, in part, involves δ subunit-containing GABA_A_Rs.

## Materials and Methods

### Animals

Adult male and female C57BL/6J mice, 8–12 weeks of age, were purchased from The Jackson Laboratory (stock #000664) and group housed in temperature- and humidity-controlled housing rooms on a 12 h light/dark cycle (lights on at 7:00 A.M.) with *ad libitum* food and water. Animals were handled according to protocols and procedures approved by the Tufts University Institutional Animal Care and Use Committee. Female mice are maintained in an acyclic state without exposure to males. Global *Gabrd*^−/−^ knock-out mice were bred in house (Mihalek et al., 1999, 2001). Mice were single housed and habituated to new cages for 24 h before the start of experiments.

### Stereotaxic surgery

All mice undergoing surgery were anesthetized with ketamine/xylazine (90 and 5–10 mg/kg, i.p., respectively) and treated with sustained release buprenorphine (0.5–1.0 mg/kg, s.c.). A lengthwise incision was made to expose the skull and a unilateral craniotomy was performed to lower a depth electrode [paraformaldehyde (PFA)-coated stainless steel wire, A-M Systems] into the BLA [anteroposterior (AP), −1.50 mm; mediolateral (ML), −3.30 mm; dorsoventral (DV), −5 mm], affixed to a head mount (catalog #8201, Pinnacle) with stainless steel screws as ground, reference, and frontal cortex EEG (AP, +0.75 mm; ML, ±0.3 mm; DV, −2.1 mm) electrodes. EMG wires were positioned in the neck muscles.

### LFP recordings

LFP recordings were performed in male and female C57BL/6J and *Gabrd*^−/−^ mice after a week of recovery from implant surgery. LFP recordings were acquired using Lab Chart software (AD Instruments) collected at 4 kHz and amplified 100×. Spectral analysis was performed in MATLAB (Antonoudiou et al., 2021) using MatWAND (https://github.com/pantelisantonoudiou/MatWAND), which uses the fast Fourier transform, similar to previous reports (Kruse and Eckhorn, 1996; Frigo and Johnson, 1998; Pape et al., 1998; Freeman et al., 2000). Briefly, recordings were divided into 5 s overlapping segments and the power spectral density for a range of frequencies was obtained (Oppenheim et al., 1999). LFP power was quantified as a power area.

### Acute and repeated alcohol exposure

Mice were habituated to new cages with *ad libitum* food and water for 24 h before starting the experimental paradigm. All injections were performed 2–3 h into the light cycle (on at 7:00 A.M.) at the same time each day across all cohorts. A dose response of four doses (0.5, 1.0, 1.5, and 2.0 g/kg, i.p.) was completed in a group of male C57BL/6J mice to determine the appropriate dose to use in our experiments. The acute exposure consisted of a 60 min baseline period followed by a saline injection (0.9% NaCl, i.p.) and a subsequent 1 g/kg ethanol injection [20% (v/v), i.p.]. The repeated exposure consisted of a 60 min baseline period followed by an intraperitoneal injection of either saline or 1 g/kg ethanol [20% (v/v)] for 5 consecutive days. For the females, the acute ethanol exposure was calculated from the first day of the repeated exposure paradigm.

### Blood ethanol concentration measurements

Blood from the submandibular vein was collected from a separate cohort of male and female C57BL/6J mice 15 min after exposure to alcohol (1 g/kg, i.p.) on days 1, 2, and 5 of repeated alcohol exposure. Blood was spun down at 1.8 × *g* for 15 min at 4°C, and serum was stored at −80°C until blood ethanol concentration (BEC) measurements were performed using the BioAssay Systems EnzyChrom Ethanol Assay Kit (ECET-100) according to the manufacturing protocol. Measurements are reported in milligrams per deciliter.

### Immunohistochemistry

Immunohistochemistry was performed as previously reported (Melón et al., 2019) in a separate cohort of C57BL/6J mice 30 min following repeated exposure to vehicle or alcohol for 5 d. Mice were anesthetized with isoflurane, transcranially perfused with 0.9% saline and 4% PFA, the brains were rapidly excised, fixed in 4% PFA overnight, and subsequently cryoprotected in 10% and 30% sucrose. The brains were then flash frozen using isopentane and stored at −80°C until cryosectioning. Free-floating 40 μm coronal slices were costained for PV and δ using universal antigen retrieval buffer (catalog #CTS015, R&D Systems) and primary antibodies against δ-GABA_A_R (1:100; catalog #868A-GDN, Phosphosolutions) and PV (1:1000; catalog #P3088, Sigma-Aldrich) for 72 h at 4°C. The slices were then incubated with a biotinylated goat anti-rabbit (1:1000; catalog #BA1000, Vector Laboratories) and Alexa-Fluor 647-conjugated goat anti-mouse (1:200; catalog #A28181, Thermo Fisher Scientific) for 2 h at room temperature and streptavidin-conjugated Alexa-Fluor 488 (1:200; catalog #S32354, Thermo Fisher Scientific) for 2 h at room temperature. Slices were mounted and coverslipped with antifade hard set mounting medium with DAPI (VECTASHIELD, catalog #H1500, Vector Laboratories). Fluorescent labeling in the BLA was imaged on a Nikon A1R confocal microscope, and *z*-stacks were acquired using a 20× objective. Camera settings were kept consistent across samples and cohorts. The images were analyzed using ImageJ software by outlining PV-positive interneurons using the ROI manager and measuring the integrated density of PV and δ expression on the outlined PV-positive interneurons. Each cell was considered its own data point within each animal.

### Statistical analysis

Data were analyzed using Prism 8 software (GraphPad Software) and MatWAND in MATLAB (MathWorks). To ensure a consistent time period for analysis across cohorts, we analyzed the first 40 min of baseline and the first 35 min of each injection period. Repeated-measures two-way ANOVAs were performed to detect the significance of frequency, treatment, sex, or genotype. A Greenhouse–Geisser correction was applied where necessary. A mixed-effects model was used if values were missing across days. A *post hoc* Sidak’s multiple-comparison test was performed to identify significant differences of specific frequency ranges. ANOVA results are reported in Extended Data Tables 1-1, 2-1, and 3-1. Multiple comparisons are reported in Extended Data 1-2, 2-2, and 3-2. *p* values < 0.05 were considered significant. All *n* values for each treatment group are shown in the figure legends.

10.1523/ENEURO.0010-22.2022.tab1-1 Table 1-1 Summary of ANOVAs for acute alcohol experiments in C57BL/6J mice. Download Table 1-1, DOCX file.

10.1523/ENEURO.0010-22.2022.tab1-2 Table 1-2 Summary of multiple-comparison tests for acute alcohol experiments in C57BL/6J mice. Download Table 1-2, DOCX file.

10.1523/ENEURO.0010-22.2022.tab2-2 Table 2-2 Summary of multiple-comparison tests for acute alcohol experiments in *Gabrd^– /–^* mice. Download Table 2-2, DOCX file.

10.1523/ENEURO.0010-22.2022.tab3-2 Table 3-2 Summary of multiple-comparison tests for repeated alcohol experiments. Download Table 3-2, DOCX file.

## Results

### Alcohol modulates BLA network states

**Figure 1.**
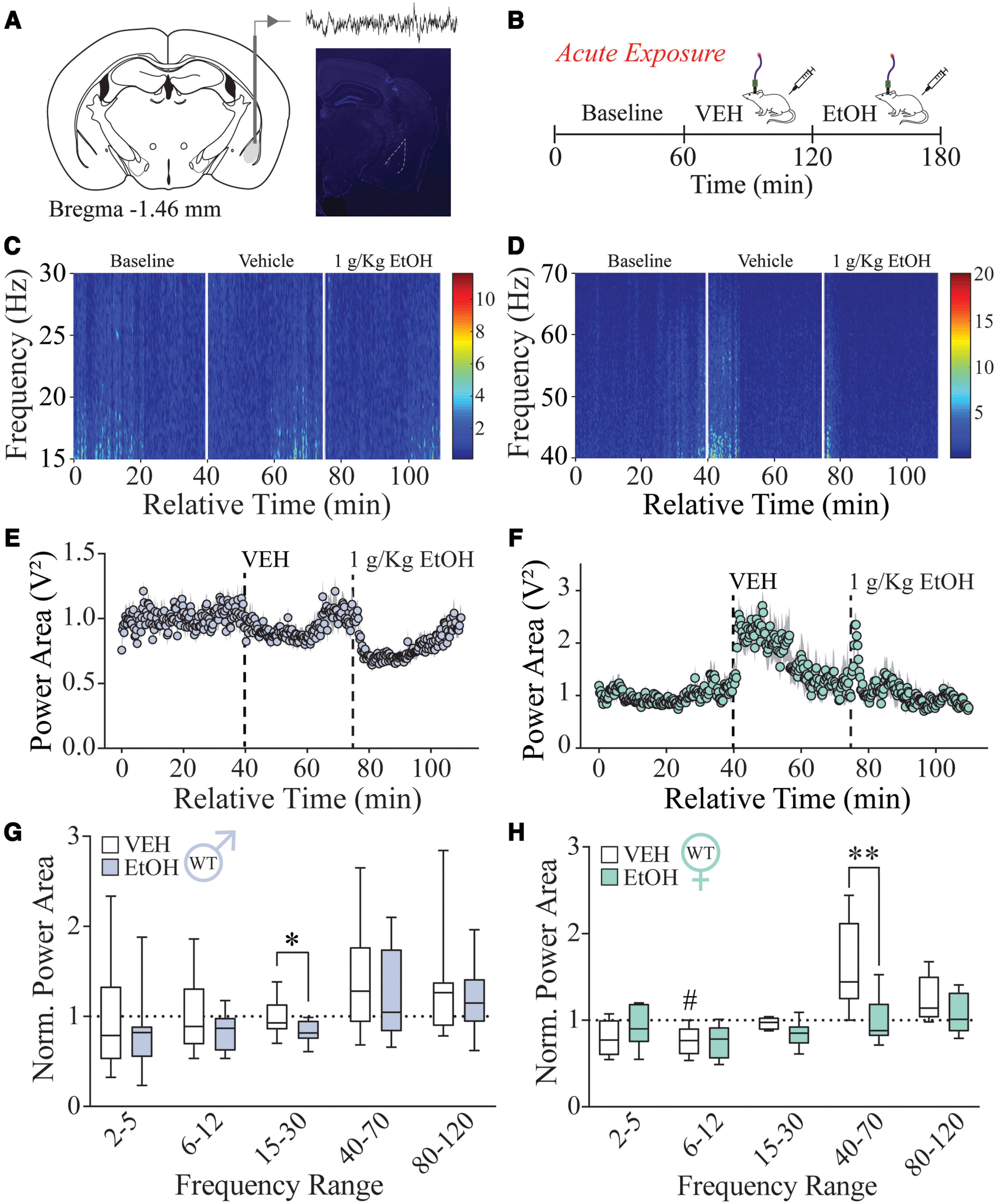
Acute alcohol exposure alters BLA network activity differently in male and female C57BL/6J mice. ***A***, Representative targeting of BLA LFP recordings. ***B***, Acute alcohol exposure paradigm consisted of LFP recordings during baseline (60 min), vehicle injection (0.9% saline, i.p.; 60 min), and a treatment injection (0.9% saline or 1 g/kg alcohol, i.p.; 60 min). This dose was determined through a dose response (Extended Data Fig. 1-2). ***C***, ***D***, Representative male spectrogram of normalized beta power (15–30 Hz; ***C***) and representative female spectrogram of normalized gamma power (40–70 Hz; ***D***) from acute alcohol exposure. ***E***, ***F***, Average normalized beta power in males (***E***) and average normalized gamma power in females (***F***) during acute injections of vehicle and alcohol (1 g/kg, i.p.; Extended Data Fig. 1-2, dose response). Dots represent the mean, and the shaded region represents the SEM. ***G***, ***H***, Normalized power area for vehicle/alcohol acute exposure in males (*n *=* *11; ***G***) and females (*n *=* *8; ***H***). #*p *<* *0.05 vs baseline; *p <* *0.05, **p <* *0.01 vs vehicle. Acute vehicle exposure does not alter BLA network activity in C57BL/6J mice (Extended Data Fig. 1-1). Summaries of ANOVA and multiple-comparison tests can be found in Extended Data Tables 1-1 and 1-2.

To characterize the effect of acute alcohol on BLA oscillations in wild-type mice, we recorded LFPs in the BLA of C57BL/6J mice in response to either a vehicle (0.9% saline, i.p.) or alcohol (1 g/kg, i.p.) injection (Fig. 1*A*,*B*). We found that vehicle injections in male C57BL/6J mice significantly decreased high theta power (6–12 Hz; *p *=* *0.0117; 95% CI = 0.04658, 0.4029), while increasing the low gamma power (40–70 Hz; *p *=* *0.0027; 95% CI = −0.9799, −0.2066), and high gamma power (80–120 Hz; *p *=* *0.0103; 95% CI = −0.6491, −0.08079) compared with baseline (Extended Data Fig. 1-1*A*). However, we did not find any difference between the two vehicle injections, indicating there was no sensitization or adaptation to the second injection. We have previously observed the impact of vehicle injections on oscillatory states in the BLA (Antonoudiou et al., 2021), which likely reflects the network response to the stress of the injection. Therefore, all results are compared with the first vehicle injection within the treatment paradigm.

10.1523/ENEURO.0010-22.2022.f1-1 Figure 1-1 Acute vehicle exposure does not alter BLA network activity in C57BL/6J mice. ***A***, ***B***, Normalized power area for vehicle/vehicle acute exposure in male (*n *=* *13; ***A***) and female (*n *=* *6; ***B***) mice. ***C***, ***D***, Power spectral density of baseline, vehicle, and 1 g/kg alcohol injection over 0–80 Hz in male mice (***C***) and female mice (***D***). #*p *<* *0.05, ##*p *<* *0.01 versus baseline. Download Figure 1-1, TIF file.

**Figure 2.**
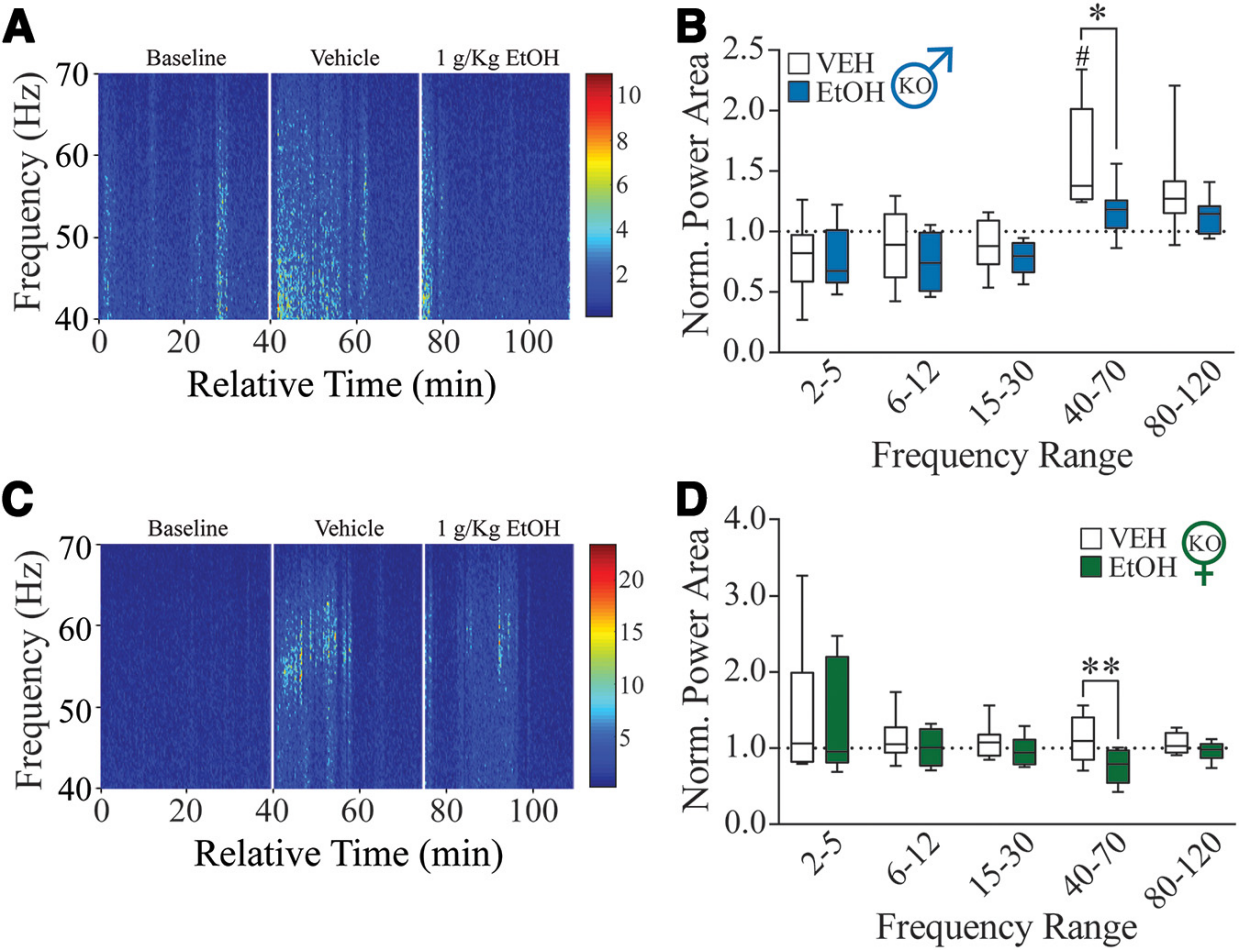
Acute alcohol exposure produces effects in *Gabrd*^−/−^ mice similar to those in female C57BL/6J mice. ***A***, ***C***, Representative male (***A***) and female (***C***) spectrogram of normalized gamma power from acute alcohol exposure. ***B***, ***D***, Normalized power area for vehicle/alcohol acute exposure in male (*n *=* *9; ***B***) and female (*n *=* *8; ***D***) *Gabrd*^−/−^ mice. #*p *<* *0.05 versus baseline; *p <* *0.05, **p <* *0.01 versus vehicle. Acute vehicle exposure does not alter BLA network activity in *Gabrd*^−/−^ mice (Extended Data Fig. 2-1). Summaries of ANOVA and multiple-comparison tests can be found in Extended Data Tables 2-1 and 2-2.

10.1523/ENEURO.0010-22.2022.f1-2 Figure 1-2 Effects of an alcohol dose response on BLA network states. ***A***, Power area difference of 2–5, 6–12, and 15–30 Hz frequency ranges across alcohol doses in male C57BL/6J mice (0.5, *n *=* *8; 1.0, *n *=* *8; 1.5, *n *=* *4; 2.0, *n *=* *7). ***B–F***, Shaded region indicates doses that caused high immobility in mice. Normalized power area for vehicle/alcohol exposure across doses for 2–5 Hz (***B***), 6–12 Hz (***C***), 15–30 Hz (***D***), 40–70 Hz (***E***), and 80–120 Hz (***F***). *p <* *0.05, **p <* *0.01 versus vehicle. Download Figure 1-2, TIF file.

We performed a dose response examining changes in network activity in response to four different doses of alcohol (0.5, 1.0, 1.5, 2.0 g/kg, i.p.; Extended Data Fig. 1-2). These experiments determined that the 1.0 g/kg dose was capable of significantly altering relevant BLA network state frequencies without producing lethargy or sedation in the mice (6–12 Hz: *p *=* *0.030; 95% CI = 0.024, 0.450; Extended Data Fig. 1-2*A–C*). Therefore, we chose this concentration for our experiments throughout this study. In response to alcohol treatment in male C57BL/6J mice, the power in the beta frequency range (15–30 Hz) is decreased compared with vehicle (*p *=* *0.034; 95% CI = 0.01033, 0.2925; Fig. 1*C*,*E*,*G*, Extended Data Fig. 1-1*C*). This indicates that alcohol can modulate specific oscillatory frequencies within the BLA that are implicated both in addiction and mood disorders (Jurado-Barba et al., 2020).

### Alcohol modulates BLA network states in a sex-dependent manner

Because of the well documented sex differences in alcohol related behaviors (Melón et al., 2013; Barkley-Levenson and Crabbe, 2015; Becker and Koob, 2016; Sneddon et al., 2019), we treated female C57BL/6J to the same acute alcohol paradigm as described in males (Fig. 1*B*). Similar to the males, we did not find any significant differences between the two vehicle injections in the vehicle/vehicle control experiments in females (Extended Data Fig. 1-1*B*). We did find that vehicle significantly decreased high theta power compared with baseline (*p *=* *0.0273; 95% CI = 0.2699, 0.4296; Fig. 1*H*), similar to what we observed in the males. Additionally, there was no significant difference between the male and female C57BL/6J BLA LFP response to the vehicle injection (figure not shown).

In response to acute alcohol exposure, we found that alcohol significantly decreased the gamma-band power in female C57BL/6J mice compared with vehicle (*p *= 0.0014; 95% CI = 0.2955, 0.9441; Fig. 1*D*,*F*,*H*, Extended Data Fig. 1-1*D*), a unique signature from the males. Interestingly, this reduction in gamma power represents a blunting of the increase in power exhibited by the vehicle injection (Fig. 1*F*). Although alcohol decreased BLA power in different frequency bands in males and females, there were no direct significant differences between groups (figure not shown). Collectively, these data suggest that acute ethanol modulates the BLA network differently in male and female mice.

### Alcohol modulation of BLA network states involves δ subunit-containing GABA_A_Rs

Previous literature has supported the role of δ-GABA_A_Rs in mediating the effects of alcohol on tonic inhibition, drinking, and withdrawal behaviors (Wallner et al., 2003; Santhakumar et al., 2007; Darnieder et al., 2019; Melón et al., 2019). Therefore, to test whether alcohol is mediating its effects on BLA network states through δ-GABA_A_Rs, we repeated the same procedure in male and female *Gabrd*^−/−^ mice. We found that vehicle injections significantly increased BLA power at low gamma frequencies only in the vehicle/alcohol condition in male *Gabrd*^−/−^ mice compared with baseline (*p *=* *0.0121; 95% CI = −1.062, −0.1299; Fig. 2*A*,*B*). In both *Gabrd*^−/−^ males and females, we did not find any significant difference between vehicle injections (Extended Data Fig. 2-1).

10.1523/ENEURO.0010-22.2022.f2-1 Figure 2-1 Acute vehicle exposure does not alter BLA network activity in *Gabrd^– /–^* mice. ***A***, ***B***, Normalized power area for vehicle/vehicle exposure in male mice (*n *=* *8; ***A***) and female mice (*n *=* *7; ***B***). Download Figure 2-1, TIF file.

Unlike C57BL/6J males, acute alcohol significantly decreased the low gamma band of *Gabrd*^−/−^ males (*p *=* *0.020; 95% CI = 0.07, 0.78; Fig. 2*A*,*B*) and *Gabrd*^−/−^ females (*p *=* *0.0012; 95% CI = 0.1731, 0.5352; Fig. 2*C*,*D*) compared with vehicle. This effect was similar to, but not as robust an effect as, that in C57BL/6J females. However, direct comparisons between male C57BL/6J and male *Gabrd*^−/−^ mice or between C57BL/6J females and *Gabrd*^−/−^ females did not detect significant differences in the ability of alcohol to modulate oscillatory states (figure not shown). Collectively, these data suggest that the loss of the GABA_A_R δ subunit impacts the network effect of alcohol more profoundly in males and induces a similar network effect as that observed in C57BL/6J females.

### Ability of repeated alcohol exposure to modulate BLA network states is dependent on δ subunit-containing GABA_A_Rs

**Figure 3.**
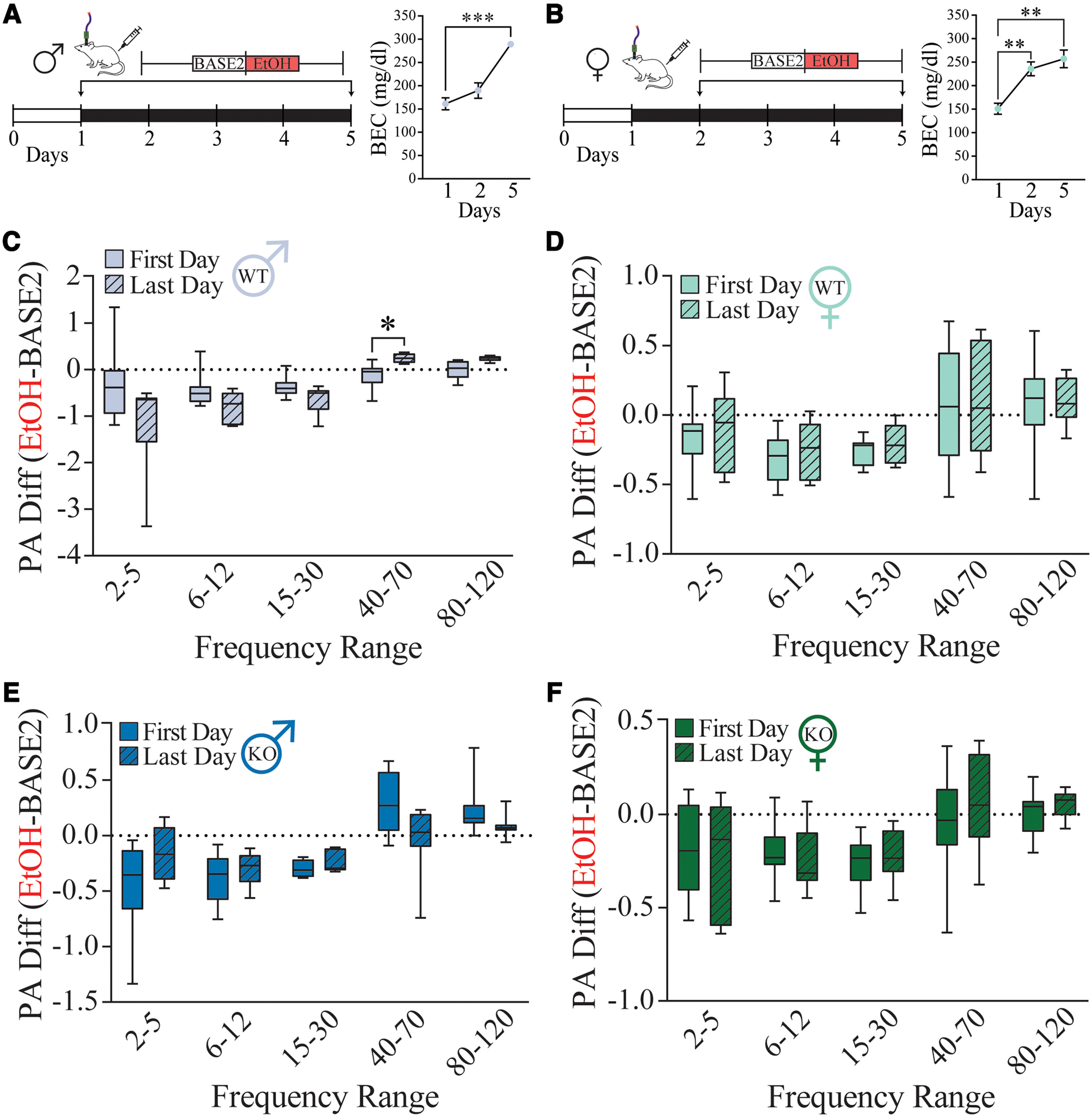
Repeated alcohol exposure exaggerates BLA network modulation in male C57BL/6J mice. ***A***, Experimental paradigm (left) of the repeated alcohol exposure procedure in male C57BL/6J and *Gabrd*^−/−^ mice of BLA LFP recordings during baseline (60 min) and vehicle or alcohol injections (0.9% saline or 1 g/kg alcohol, i.p.; 60 min) over 5 d. BEC measurements (right) of male C57BL/6J mice (*n *=* *6) taken 15 min after alcohol exposure (1 g/kg, i.p.) on days 1, 2, and 5 of exposure. ***p <* *0.0001. ***B***, Repeated alcohol exposure paradigm (left) for female C57BL/6J and *Gabrd*^−/−^ mice, which includes the acute alcohol exposure and the repeated alcohol exposure as day 2 (first day) to 5 (last day). Justification for using day 2 instead of day 1 is in Extended Data Figure 3-3. BEC measurements (right) of female C57BL/6J mice (*n *=* *5) taken 15 min after alcohol exposure (1 g/kg, i.p.) on days 1, 2, and 5 of exposure. ***C***–***F***, Change in the effect of alcohol on the first and last days of exposure in male (first day, *n *=* *8; last day, *n *=* *6; ***C***) and female (first day, *n *=* *10; last day, *n *=* *8; ***D***) C57BL/6J mice and male (first day, *n *=* *8; last day, *n *=* *8; ***E***) and female (first day, *n *=* *8; last day, *n *=* *7; ***F***) *Gabrd*^−/−^ mice. *p <* *0.05 versus first exposure. Repeated vehicle exposure does not change BLA network activity (Extended Data Fig. 3-1). Baseline network activity is modulated by repeated alcohol exposure in male C57BL/6J mice (Extended Data Fig. 3-2). Summaries of ANOVA and multiple-comparison tests can be found in Extended Data Tables 3-1 and 3-2.

Since we established that acute alcohol could modulate specific oscillatory frequencies in the BLA, we were interested in how BLA LFPs changed over time in response to repeated doses of alcohol. Male C57BL/6J and *Gabrd*^−/−^ mice received vehicle (0.9% saline) or low dose (1 g/kg, i.p.) alcohol for 5 consecutive days (Fig. 3*A*). We did not find significant effects of repeated vehicle injections across days in either the male C57BL/6J or male *Gabrd*^−/−^ mice (Extended Data Fig. 3-1*A*,*C*).

10.1523/ENEURO.0010-22.2022.f3-2 Figure 3-2 Repeated alcohol exposure modulates baseline network activity in male C57BL/6J mice. ***A***, ***B***, Change in baseline on the first and last day of alcohol exposure in male (***A***) and female (***B***) C57BL/6J mice and male (***C***) and female (***D***) *Gabrd^– /–^* mice. *p <* *0.05. Download Figure 3-2, TIF file.

10.1523/ENEURO.0010-22.2022.f3-3 Figure 3-3 Effects of acute alcohol exposure on day 1 (acute) and day 5 do not differ in female *Gabrd^– /–^* mice. ***A***, Normalized power area of alcohol injections during the acute alcohol injection and day 2 in female C57BL/6J mice (*n *=* *10). ***B***, Normalized power area of alcohol injections during the acute alcohol injection and day 2 in female *Gabrd^– /–^* mice. ***B***, Normalized power area of alcohol injections during acute alcohol injection and day 5 in female *Gabrd^– /–^* mice. **p <* *0.01. Download Figure 3-3, TIF file.

10.1523/ENEURO.0010-22.2022.tab2-1 Table 2-1 Summary of ANOVAs for acute alcohol experiments in *Gabrd^– /–^* mice. Download Table 2-1, DOCX file.

10.1523/ENEURO.0010-22.2022.f3-1 Figure 3-1 Repeated vehicle exposure does not change BLA network activity in C57BL6/J and *Gabrd^– /–^* mice. ***A***–***D***, Power area difference between vehicle and baseline for the first and last days of exposure in male (first day, *n *=* *4; last day, *n *=* *3; ***A***) and female (first day, *n *=* *5; last day, *n *=* *6; ***B***) C57BL/6J mice and male (first day, *n *=* *4; last day, *n *=* *4; ***C***) and female (first day, *n *=* *7; last day, *n *=* *7; ***D***) *Gabrd^– /–^* mice. Download Figure 3-1, TIF file.

Interestingly, in response to repeated alcohol treatment, we found a change in the baseline low gamma power from the first to last day (BASE2-BASE1) in male C57BL/6J mice (*p *=* *0.0252; 95% CI = 0.03785, 0.6384; Extended Data Fig. 3-2*A*), which may be an anticipatory change associated with repeated alcohol administration. In response to alcohol treatment, we observed a significant increase in low gamma power from the first to the last day of exposure (EtOH-BASE2; *p *=* *0.0305; 95% CI = −0.7087, −0.03272; Fig. 3*C*) along with an increase in BEC (first, 161.4 mg/dl; last, 189.8 mg/dl; *p *<* *0.0001; 95% CI = −160.4, −95.81; Fig. 3*A*). In contrast, we did not observe significant effects of repeated alcohol on baseline or treatment in male *Gabrd*^−/−^ mice (Fig. 3*D*, Extended Data Fig. 3-2*C*).

**Figure 4.**
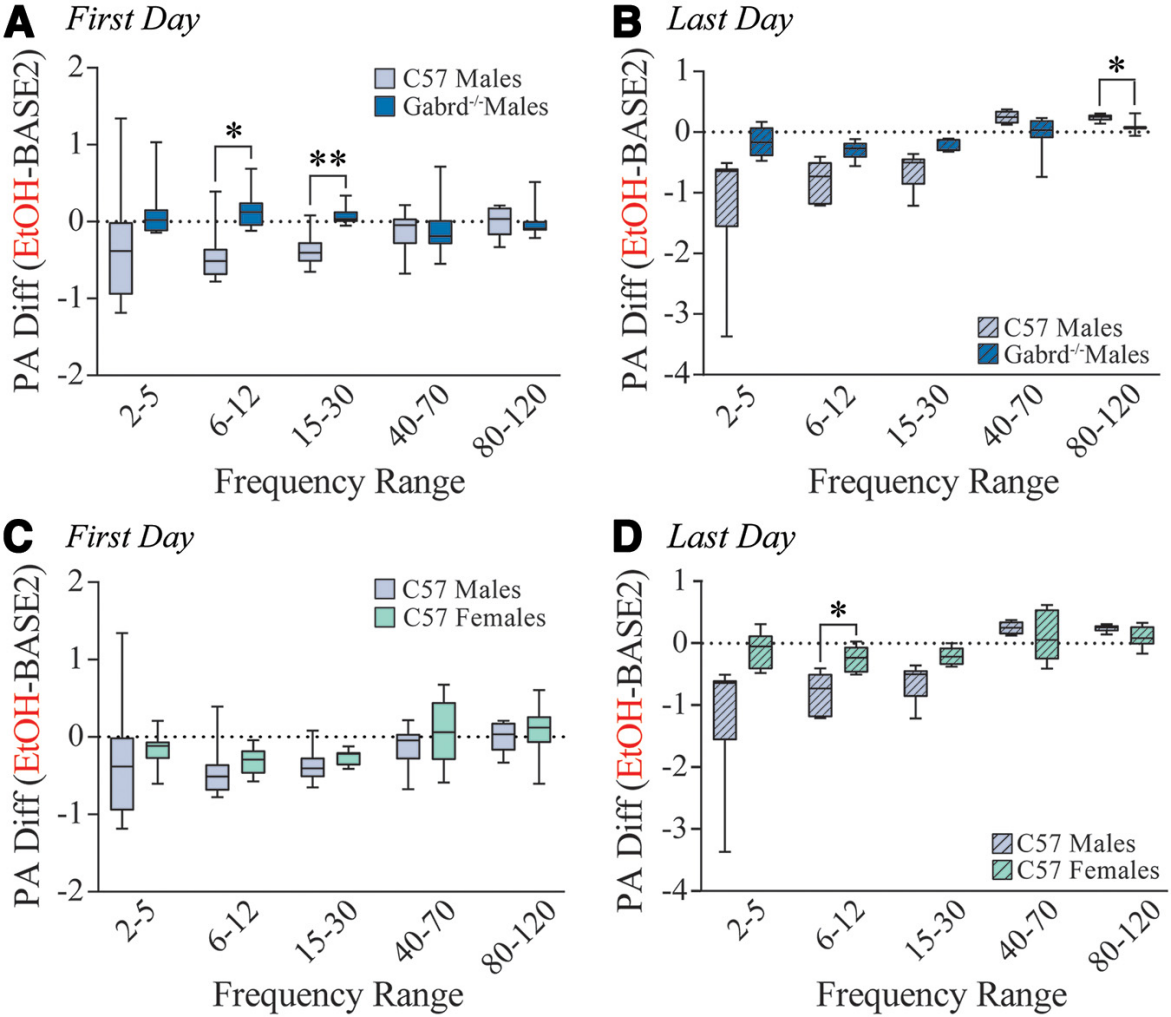
δ-GABA_A_R and sex-dependent effects of repeated alcohol exposure on BLA network activity. ***A***, ***B***, Comparison between male C57BL/6J and *Gabrd*^−/−^ mice in the response to alcohol on the first day (***A***) and last day (***B***) of exposure. ***C***, ***D***, Comparison between male and female C57BL/6J mice in the response to alcohol on the first day (***C***) and the last day (***D***) of exposure. *p <* *0.05, **p <* *0.01. Repeated alcohol has δ-GABA_A_R and sex-dependent effects on baseline network activity (Extended Data Fig. 4-1).

Direct comparison between male C57BL/6J and *Gabrd*^−/−^ mice on the first day of alcohol exposure does not reveal any significant changes within baseline (Extended Data Fig. 4-1*A*), but did find that male C57BL/6J mice had significantly decreased high theta (*p *=* *0.0138; 95% CI = −1.061, −0.1088) and beta (*p *=* *0.0022; 95% CI = −0.7193, −0.1659) BLA power compared with male *Gabrd*^−/−^ mice in response to alcohol exposure (Fig. 4*A*). By the last day, there were significant decreases within the baseline period specifically in the low gamma (*p *=* *0.0343; 95% CI = −0.9892, −0.03576) and high gamma (*p *=* *0.0062; 95% CI = −0.5908, −0.09553) BLA power in male C57BL/6J mice compared with male *Gabrd*^−/−^ mice (Extended Data Fig. 4-1*B*), again likely attributed to the role of the GABA_A_R δ subunit in the anticipatory effects of repeated alcohol exposure. In response to repeated alcohol administration, we observed a significant increase in the high gamma frequency range in male C57BL/6J mice compared with male *Gabrd*^−/−^ mice (*p *=* *0.0203; 95% CI = 0.02211, 0.2899; Fig. 4*B*). Overall, these results suggest a blunted impact of acute and repeated alcohol exposure on BLA oscillatory states in mice lacking the GABA_A_R δ subunit. Further, these data indicate a role for δ-GABA_A_Rs in adapting to alcohol exposure over time, as well as anticipating alcohol treatment, as shown by the changes in baseline in male C57BL/6J mice, but not *Gabrd*^−/−^ mice.

10.1523/ENEURO.0010-22.2022.tab3-1 Table 3-1 Summary of ANOVAs for repeated alcohol experiments. Download Table 3-1, DOCX file.

10.1523/ENEURO.0010-22.2022.f4-1 Figure 4-1 δ-GABA_A_Rs and sex-dependent effects of baseline network modulation after repeated alcohol. ***A***, ***B***, Comparison between male C57BL/6J and *Gabrd^– /–^* mice in the change in baseline on the first day (***A***) and last day (***B***) of exposure. ***C***, ***D***, Comparison between male and female C57BL/6J mice in the change in baseline on the first day (***C***) and last day (***D***) of exposure. *p <* *0.05. Download Figure 4-1, TIF file.

### Sex differences in BLA network states in response to repeated alcohol exposure

Repeated alcohol exposure in female C57BL/6J and *Gabrd*^−/−^ mice involved acute alcohol or vehicle exposure on day 1 and the repeated alcohol exposure on days 2–5 (Fig. 3*B*). We will be using their second day of exposure in our repeated alcohol comparisons, which were not significantly different in female C57BL/6J mice (Extended Data Fig. 3-3*A*). Neither day 1 nor day 2 were significantly different from day 5 in female *Gabrd*^−/−^ mice (Extended Data Fig. 3-3*B*,*C*). Therefore, we continued to use day 2 as the first day of repeated exposure in our analysis.

We did not observe significant effects of vehicle exposure across days in female C57BL/6J (Extended Data Fig. 3-1*B*) or female *Gabrd*^−/−^ mice (Extended Data Fig. 3-1*D*). Interestingly, unlike the males, we did not observe any significant effect of repeated alcohol administration in C57BL/6J female and *Gabrd*^−/−^ females across days (Fig. 3*D*,*F*, Extended Data Fig. 3-2*B*,*D*) despite an increase in BEC in female C57BL/6J mice from the first to last day of exposure (first, 151.1 mg/dl; last, 257.3 mg/dl; *p *=* *0.0072; 95% CI = −166.0, −46.32; Fig. 3*B*).

Direct comparison between C57BL/6J males and females did not reveal significant differences at any frequency range within the baseline period (Extended Data Fig. 4-1*C*) or in the effect of alcohol from baseline (Fig. 4*C*) on the first day of exposure. However, by the last day, we found an increase in high theta power (*p *=* *0.0435; 95% CI = 0.01858, 1.216) and a decrease in high gamma power within the baseline of males, but not in the females (*p *=* *0.0122; 95% CI = −0.5198, −0.06395; Extended Data Fig. 4-1*D*). In response to repeated alcohol exposure, males exhibited a significantly reduced power in the high theta frequency range in C57BL/6J males with no effect in females (*p *=* *0.0433; 95% CI = −1.085, −0.01596; Fig. 4*D*). There was no significant difference between male and female BECs on day 1 or day 5, suggesting the same level of alcohol intoxication modulates BLA LFPs differentially in the two sexes. Collectively, these data suggest that male C57BL/6J mice are becoming more sensitive to the sedative effects of alcohol as demonstrated by the significant effects of repeated alcohol administration on gamma frequency ranges, an effect that involves the GABA_A_R δ subunit (Pian et al., 2008).

### Repeated alcohol exposure alters δ expression on PV interneurons in the BLA

**Figure 5.**
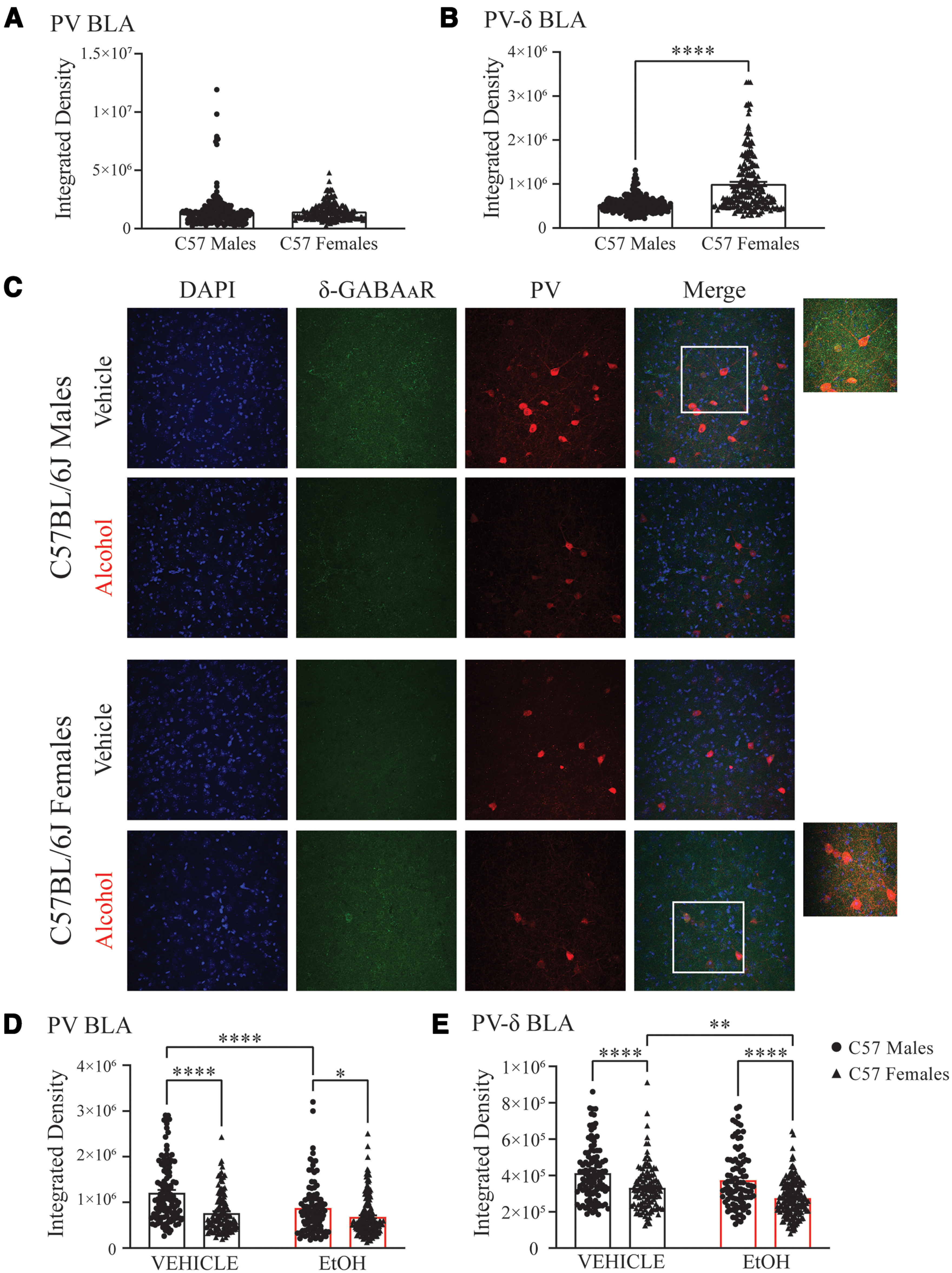
Repeated alcohol exposure reduces δ-GABA_A_R expression on PV interneurons in female C57BL/6J mice. ***A***, ***B***, Integrated density of PV immunoreactivity (***A***) and δ expression (***B***) on PV interneurons in naive male (cell, *n *=* *224; animal, *n *=* *4) and female (cell, *n *=* *202; animal, *n *=* *4) C57BL/6J mice. ***C***, Representative images from male and female C57BL/6J mice who received repeated administration of vehicle or alcohol. ***D***, ***E***, Integrated density of PV immunoreactivity (***D***) and δ expression (***E***) on PV interneurons in the BLA of male (vehicle: cell, *n *=* *112; animal, *n *=* *3; alcohol: cell, *n *=* *97; animal, *n *=* *3) and female (vehicle: cell, *n *=* *117; animal, *n *=* *3; alcohol: cell, *n *=* *169; animal, *n *=* *3) C57BL/6J mice who received repeated administrations of vehicle or alcohol. *p <* *0.05, **p <* *0.01, ****p <* *0.0001.

Alcohol exposure can change the expression of GABA_A_R subunits (Liang et al., 2004; Olsen and Spigelman, 2012; Lindemeyer et al., 2014; Follesa et al., 2015), and sex differences in GABA_A_R δ subunit expression has been reported (Maguire et al., 2005). Changes in the expression of the GABA_A_R δ subunit, whether through genetic deletions or hormone fluctuations during pregnancy, can alter specific oscillation frequencies in the hippocampus (Ferando and Mody, 2013, 2015). Therefore, we hypothesized that altered expression of the GABA_A_R δ subunit on PV interneurons in the BLA may contribute to our observed sex differences in BLA network states. Thus, we examined whether there were any potential sex differences in GABA_A_R δ subunit expression in the BLA or in δ expression associated with alcohol exposure. We observed a higher δ expression on PV interneurons in naive female C57BL/6J mice (mean* *=* *1,002,542; SEM* *=* *45,011) compared with naive male C57BL/6J mice (mean* *=* *538,252; SEM* *=* *12,440; *t*_(424)_ = 10.39, *p* < 0.0001; Fig. 5*B*) with no change to PV immunoreactivity (female: mean* *=* *1,464,808; SEM* *=* *51,507; male: mean* *=* *1,448,517; SEM* *=* *97,320; Fig. 5*A*). Interestingly, vehicle treatment alone reduced PV immunoreactivity in females compared with males (*p *<* *0.0001; 95% CI = 267746, 621366; Fig. 5*D*) and also reduced δ expression on PV neurons in females compared with males (*p *<* *0.0001; 95% CI = 35,568, 126,727; Fig. 5*E*). These data demonstrate baseline sex differences in the expression and lability of GABA_A_R δ expression on PV interneurons in the BLA.

Repeated alcohol treatment reduced PV immunoreactivity in males compared with vehicle (*p *<* *0.0001; 95% CI = 147,380, 518,402; Fig. 5*D*). In contrast, repeated alcohol exposure in females did not alter PV immunoreactivity but did significantly reduce GABA_A_R δ expression on PV interneurons compared with vehicle (*p *=* *0.0018; 95% CI = 15,583, 98,517; Fig. 5*E*), an effect that was not observed in males.

Comparing males and females exposed to repeated alcohol, PV immunoreactivity (*p *=* *0.0195; 95% CI = 22,163, 362,911; Fig. 5*D*) and δ expression on PV interneurons are reduced in females compared with males (*p *<* *0.0001; 95% CI = 54,848, 142,689; Fig. 5*E*). These data imply that changes in GABA_A_R δ expression on PV interneurons may mediate sex differences and the response to repeated alcohol exposure.

## Discussion

Network states have been shown to correlate with behavioral states, and accumulating evidence demonstrates that signature oscillatory states in the BLA are associated with fear and anxiety states (Likhtik et al., 2014; Stujenske et al., 2014; Davis et al., 2017; Antonoudiou et al., 2021). In fact, optogenetically driving specific oscillatory states influences the behavioral expression of fear (Ozawa et al., 2020) and learned helplessness (Antonoudiou et al., 2021). However, limited studies have examined the physiological, pathological, or pharmacological mechanisms mediating transitions between network and behavioral states. Recent work has demonstrated that chronic stress can perturb oscillations in the BLA and a clinically effective antidepressant treatment can restore the “healthy” network state (Antonoudiou et al., 2021; Walton et al., 2022). Here, we examine the impact of alcohol on BLA network states. Given the anxiolytic effects of alcohol, we posited that alcohol may be capable of shifting the network state toward the anxiolytic state. We demonstrate that acute alcohol exposure is capable of altering BLA network states and that there are sex differences in the effect of alcohol on BLA network states, affecting different frequencies in males and females. These data are the first to demonstrate that alcohol is capable of modulating network states associated with affective states.

It has been demonstrated that PV interneurons are critical in orchestrating oscillatory states in the BLA (Antonoudiou et al., 2021). PV interneurons in the BLA express a high density of δ-GABA_A_Rs, which have been suggested to be a target for low-dose alcohol (Sundstrom-Poromaa et al., 2002; Wallner et al., 2003; Hanchar et al., 2006; Santhakumar et al., 2007). However, the actions of alcohol directly on these receptors remains somewhat controversial (Borghese et al., 2006; Korpi et al., 2007). It is important to note that the majority of these studies focus solely on principal neurons; GABAergic interneurons, on the other hand, have a unique receptor subunit composition in which the δ subunit has been shown to partner with the α1 subunit and have been demonstrated to generate tonic GABAergic currents, which are highly sensitive to low concentrations of ethanol (Glykys et al., 2007). Thus, we proposed that the high expression of δ subunit-containing GABA_A_Rs on PV interneurons in the BLA may confer unique sensitivity to the effects of alcohol and, given the role of these interneurons in coordinating oscillations, may mediate the effects of alcohol on BLA network states. Here we demonstrate that δ-GABA_A_Rs influence the ability of alcohol to alter specific oscillatory states in the BLA, blunting the ability to shift network states. Specifically, we observed a reduction of beta power from acute ethanol in male wild-type mice that was blunted in mice lacking δ-GABA_A_Rs (Figs. 1, 2). Others have found reductions of beta power in the nucleus accumbens shell during alcohol relapse related to reduced synchrony of the local network, suggesting that this reduction we find in response to alcohol may also be from reduced synchrony (Hadar et al., 2017). This power detected in the beta frequency may arise from the neighboring high-theta oscillator, given the lack of a clear beta peak (Extended Data Fig. 1-1*C*). Regardless, these data suggest that δ subunit-containing GABA_A_Rs are important players in mediating the effects of alcohol on oscillatory states related to mood/anxiety, although it is also possible that other GABA_A_R subtypes are involved. Previous studies demonstrated that the δ subunit has a specific role in lower frequencies compared with higher frequencies (Antonoudiou et al., 2021), which may be true for the effects of alcohol as well. Further studies are required to investigate the impact of other GABA_A_R subtypes in mediating the ability of alcohol to modulate BLA network states given that previous studies have implicated other GABA_A_R subtypes, such as the γ2 subunit, in anxiety-like behavior (Chandra et al., 2005) and alcohol withdrawal severity (Buck and Hood, 1998). It is also possible that the indirect effects of alcohol on receptor expression, neurotransmitter availability, and other neuromodulators could account for the changes in BLA oscillations observed here (Morrow et al., 2001; Fleming et al., 2009; Olsen and Liang, 2017). For example, the effects of alcohol have been suggested to be mediated through the action of neuroactive steroids (Morrow et al., 2001; Finn et al., 2010; Finn and Jimenez, 2018), and, given recent evidence that allopregnanolone can alter BLA network states (Antonoudiou et al., 2021; Walton et al., 2022), this may be an indirect mechanism whereby alcohol could modulate BLA network states. Arguing against this indirect mechanism is the evidence that alcohol exerts unique effects on BLA network states compared with allopregnanolone (Antonoudiou et al., 2021).

Since expression of δ subunit-containing GABAARs have been shown to be sensitive to ovarian steroid hormone modulation and are implicated in sex differences in alcohol intake (Darnieder et al., 2019), we hypothesized that there may be sex differences in the ability of alcohol to modulate BLA network states through actions on these receptors. In fact, we do observe sex differences in the modulation of BLA network states by alcohol, although both male and female C57BL/6J mice reach similar BEC levels after alcohol exposure (Fig. 3*A*,*B*). Interestingly, the loss of the GABA_A_R δ subunit in males shifts the alcohol modulation of the BLA network state toward the signature that we observe for female C57BL/6J mice (Figs. 1, 3), and we believe that the observed sex differences in the expression of δ subunit-containing GABA_A_Rs in the BLA (Fig. 5) may underlie these differences. Further, there are well documented sex differences in responses to alcohol, alcohol-related anxiety-like behavior, and estrous cycle-dependent δ expression (Maguire et al., 2005; Rhodes et al., 2005; Barkley-Levenson and Crabbe, 2015), consistent with our observations of sex differences in the alcohol-induced modulation of network states. Additionally, sex differences have been reported in neural oscillations in major depressive disorder with oscillatory signatures of susceptibility (Thériault et al., 2021). Future studies are required to evaluate the relationship between the capacity of alcohol to modulate network states and voluntary alcohol consumption, the anxiolytic effects of alcohol, and the anxiogenic effects of alcohol withdrawal.

To investigate whether the effect of alcohol on network states may be altered after repeated exposure, we treated mice with low-dose alcohol for up to 5 d. Interestingly, we found that BLA network states changed before alcohol exposure. Given the evidence that the amygdala is involved in valence encoding and assignment, it is possible that network state changes before the alcohol exposure is reflective of the anticipation or expectation of the event, which has been demonstrated in a pavlovian conditioning paradigm (Pignatelli and Beyeler, 2019; Tallot et al., 2020). We found robust effects of repeated alcohol exposure on the BLA LFP response of male C57BL/6J mice, which was significantly different from the male *Gabrd*^−/−^ and female C57BL/6J mice. In fact, we found no differences in the extent of the effect of repeated alcohol on network states in female C57BL/6J or *Gabrd*^−/−^ mice despite similar BEC levels between male and female C57BL/6J mice. It is possible that the change in BLA power in male C57BL/6J mice across repeated alcohol exposure is because of the increase in BEC rather than an adaptation to the injections. Indeed, our dose response data show that higher doses of alcohol do have larger effects on BLA network states. Alcohol administration prominently affected gamma-band oscillations in the BLA, a network activity that has been associated with local network synchrony, affective learning, and memory consolidation, (Bocchio et al., 2017; Antonoudiou et al., 2021; Headley et al., 2021). Given the critical role of PV interneurons in the generation of BLA gamma oscillations (Antonoudiou et al., 2021; Headley et al., 2021), alcohol may directly modulate PV interneuron signaling. It is possible that reduction in tonic inhibition of PV interneurons in female and *Gabrd*^−/−^ mice makes PV interneurons more susceptible to the effects of acute ethanol leading to disruption in the generation of gamma network oscillations in BLA.

Since the downregulation of the δ subunit has been thought to confer tolerance to alcohol (Olsen and Liang, 2017), the reduction of δ subunit in female, but not male, C57BL/6J mice could explain the lack of effects on LFPs after repeated alcohol exposure. Further, GABA_A_R agonists and positive allosteric modulators, like neurosteroids, which exert effects through the δ subunit, can block tolerance to the sedative effects of alcohol (Debatin and Barbosa, 2006; Barbosa and Morato, 2007). This could explain why alcohol does not change the BLA network in female C57BL/6J and *Gabrd*^−/−^ mice, who have reductions in δ-GABA_A_R expression, after repeated administration like it does in male C57BL/6J mice. However, this study did not directly measure sensitization or tolerance to the effects of alcohol, and future studies could link network and behavioral changes. Last, we found that δ expression on PV interneurons is increased in naive female C57BL/6J mice compared with males. Because we did not see any baseline differences in BLA network states between male and female C57BL/6J mice, this difference in expression may not impact BLA oscillations, but the expression of δ in females does influence the response to alcohol exposure.

The literature and recent findings demonstrate a strong role for PV interneurons in oscillation generation (Antonoudiou et al., 2021), giving support to the likely fact that the effects of alcohol on PV interneurons are influencing the oscillations. However, because of the heterogeneity of the interneuron population in the BLA, it is possible that other interneuron types, like somatostatin, cholecystokinin, or PKC-δ-expressing cells may be involved in effecting oscillations (Klausberger et al., 2005). Furthermore, another major influence on BLA oscillations are other brain areas with strong network connections to the BLA, such as the mPFC, which is heavily implicated in addiction (Goldstein and Volkow, 2011; Davis et al., 2017; Ozawa et al., 2020).

Forced alcohol injections or alcohol-induced aversion can cause stress to mice, which may contribute to the observed effects (Eckardt et al., 1974). However, our experimental plan was designed to dissociate any stressful or unpleasant effects of the infusion from the effects of alcohol. Although we observed significant effects of vehicle injections on BLA LFP responses compared with baseline (Extended Data Fig. 1-1), we did not find any sensitization or tolerance to the injections in the acute alcohol experiment or across days in the repeated alcohol experiment (Extended Data Figs. 1-1, 2-1, 3-1), similar to what has been reported previously (Antonoudiou et al., 2021). Thus, we are confident that the observed effects of alcohol on BLA oscillatory states is because of the effects of alcohol rather than an aversive experience related to the route of administration. Further, our data suggest that the effects of alcohol may mitigate the stress-induced effects on the BLA network state.

To our knowledge, this is the first demonstration that alcohol can modulate oscillations in the BLA, which have been implicated in governing behavioral states. Numerous studies have investigated the relationship of BLA network states to behavioral states; however, few studies have investigated mechanisms mediating transitions between BLA network states. The current study demonstrates that alcohol can induce a transition between network states associated with fear and anxiety, which may mediate the impact of alcohol on anxiety states. Future work is required to investigate how changes in the BLA relate to other connected areas implicated in alcohol use and anxiety, such as the central amygdala, mPFC, nucleus accumbens, BNST, and ventral striatum (Janak and Tye, 2015). Recordings of oscillations are stable over long periods of time and thus can be examined throughout the addiction cycle from intoxication to withdrawal to preoccupation in specific brain areas to understand how alcohol changes communication between these areas. Thus, this novel approach may demonstrate utility in understanding the trajectory from first exposure to alcohol dependence and the contribution of both the positive and negative reinforcing effects of alcohol.

## Synthesis

Reviewing Editor: Kevin Wickman, University of Minnesota Decisions are customarily a result of the Reviewing Editor and the peer reviewers coming together and discussing their recommendations until a consensus is reached. When revisions are invited, a fact-based synthesis statement explaining their decision and outlining what is needed to prepare a revision will be listed below. The following reviewer(s) agreed to reveal their identity: John Woodward, Nicholas Gilpin.

The authors examined the effect of alcohol exposure on basolateral amygdala (BLA) network oscillations, and the role of δ-containing GABAAR specifically in parvalbumin+ neurons (PV) in the BLA, in this relationship. The rationale here is that PV cells in the BLA modulate network oscillations that are associated with fear/anxiety and PV cells express δ-containing GABAARs which are sensitive to alcohol. The authors hypothesized that alcohol modulates PV regulation of network states related to fear/anxiety via δ-containing GABAARs. To test this hypothesis, the authors used in vivo recordings of local field potentials (LFP) to determine oscillatory changes in various ranges after acute and repeated alcohol exposure in male and female wild-type C57BL/6J mice, and in global/constitutive Gabrd-/- mice. They also used IHC to determine expression of δ-containing GABAAR in PV+ neurons in the BLA in response to alcohol exposure. They found that oscillatory changes in specific ranges in response to acute alcohol differed between males and females. Also, there were inherent and alcohol-related sex differences in the activity of PV+ cells and δ-containing GABAARs on PV+ cells in BLA. The strengths of the study are the inclusion of sex differences and the study of acute vs. repeated alcohol exposure. There are a number of concerns that need to be addressed, however, to strengthen the manuscript and its conclusions:

Major comments:

1. A major issue with the manuscript is the confusing presentation of the data given the various cohorts (male, female; wild-type, knockout), treatments (vehicle, ethanol; acute, chronic), recording epochs (baseline1, baseline2, ethanol) and power spectra recorded (5 different frequency ranges) that are presented over a series of 8 multi-panel figures. This relatively confusing presentation is compounded by the modest effects reported and the somewhat inconsistent effects shown in some of the figures. While the technical aspects of the study appear sound, the significance of the findings was not readily apparent. The authors are encouraged to attempt to focus on the most relevant findings and perhaps save other, less-relevant material for supplemental figures.

2. There is no mention of blood ethanol concentrations (BECs) within or across groups or sexes. BEC measurements are essential if the goal is to correlate changes in LFP activity with the rising and falling amounts of ethanol in the brain. This is also important since the authors describe changes in LFP power that seem to reflect anticipation of the injection, in addition to the direct pharmacological effects of ethanol on BLA neural activity. Furthermore, male and female mice may experience different BECs following the same dose of ethanol. Including these data is important given that some of the sex-dependent differences in LFP following ethanol injection could stem from different levels of ethanol.

3. The IHC data shown for the delta receptor in Figure 8 is not convincing. The authors should show similar images for the delta KO mice to demonstrate the specificity of the delta antibody.

Clarifications required:

- Were different males, but the same females, used for acute and repeated exposure groups? If so, please justify. If not, please clarify.

- Why was the 1 g/kg ethanol dose chosen?

- When were tissue samples collected for IHC in comparison to the last dose of alcohol? What were BECs at this time point? Why was this time point chosen?

- The authors mention the role of ovarian hormones in drinking, mood, oscillatory state and expression of δ-GABAARs, but do not mention the cycle of the mice used in these experiments.

- What is the arrow in Figure 1A pointing at?

- Figure 1 and 2 should clearly indicate the use of male (Fig 1) and female (Fig 2) mice. In addition, Figure 2 should include a power spectrum curve as shown in Figure 1 and a dotted line should be added to Fig 2B.

- Figure 1C, text results do not match the figure. High theta is reduced, but low/high gamma are increased.

- In Figure 2D, an ethanol effect is reported, but it looks as if reduction in power area was occurring prior to ethanol treatment, and it reached asymptote with alcohol. Please address/clarify

- Line 257: How is tolerance being defined? Tolerance to which effects?

- Include the catalog number and other relevant details for antibodies

- Line 196-198: Is this comparing C57 and Gabrd animals?

Minor issues

- PV+ cells operate in the gamma frequency range, and there were several sex differences and alcohol effects observed in this frequency range. The Discussion, however, centers on overall changes in oscillatory states of the network, while most changes occurred in the gamma frequency.

- Lines 251 and 270: Re-word second sentence.

- Lines 259-269: Please strengthen the rationale for examining sex differences in PV δ-GABAAR’s regulation of network oscillations.

- Line 267: Remove the word “changes”

- The labelling of supplementary figures is confusing. There are no numbers on any figures and attachments do not specify figure or table number, making it difficult to link results to figures.

- Please proofread for typos and confusing wording

## Author Response

The authors would like to sincerely thank the reviewers for their positive review of the study and concrete steps to improve the manuscript. Based on the reviewer’s comments, we have reorganized the text and figures to make the presentation of the results clearer for the readers. We have responded to each of the reviewer’s concerns individually below. For clarity, the reviewer’s comments are in regular text and the author’s responses are in bold text.

Major comments:

1. A major issue with the manuscript is the confusing presentation of the data given the various cohorts (male, female; wild-type, knockout), treatments (vehicle, ethanol; acute, chronic), recording epochs (baseline1, baseline2, ethanol) and power spectra recorded (5 different frequency ranges) that are presented over a series of 8 multi-panel figures. This relatively confusing presentation is compounded by the modest effects reported and the somewhat inconsistent effects shown in some of the figures. While the technical aspects of the study appear sound, the significance of the findings was not readily apparent. The authors are encouraged to attempt to focus on the most relevant findings and perhaps save other, less-relevant material for supplemental figures. We thank the Reviewers for communicating that the way that the figures were initially presented was confusing. We have reorganized the figures and find the presentation of the figures much more straightforward in the revised manuscript. We condensed our figures to better convey our significant results from a total of 14 to 12 (5 main figures) including extended data figures. The figure panels that compared LFP changes between genotypes or sexes that did not show significant effects were removed. Vehicle data and baseline LFP data from the repeated alcohol experiments were added to the extended data figures to make the main figures clearer.

2. There is no mention of blood ethanol concentrations (BECs) within or across groups or sexes. BEC measurements are essential if the goal is to correlate changes in LFP activity with the rising and falling amounts of ethanol in the brain. This is also important since the authors describe changes in LFP power that seem to reflect anticipation of the injection, in addition to the direct pharmacological effects of ethanol on BLA neural activity. Furthermore, male and female mice may experience different BECs following the same dose of ethanol. Including these data is important given that some of the sex-dependent differences in LFP following ethanol injection could stem from different levels of ethanol. We performed BEC measurements in male and female C57BL/6J mice 15 minutes after alcohol administration (1 g/kg i.p.) on day one, day two, and day five of alcohol exposure. We selected the 15-minute time point because this is in the middle of our observed LFP effects which last around 30 minutes after administration. Results can be found in figure 3. We did not find significant sex differences in BEC measurements but did find an increase in BEC from day one to day five in both sexes. This information is now provided in the manuscript (pg. 6; pgs. 11-12).

3. The IHC data shown for the delta receptor in Figure 8 is not convincing. The authors should show similar images for the delta KO mice to demonstrate the specificity of the delta antibody. We, and others, have used this antibody extensively in previous studies (Agoglia et al., 2020; Lee, MacKenzie, Hooper, & Maguire, 2016; Lee, Sarkar, & Maguire, 2014; Sarkar, Wakefield, Mackenzie, Moss, & Maguire, 2011). We have previously validated the specificity of this antibody using a global Gabrd knockout mice (Gabrd-/-) (Sarkar et al., 2011) and in several conditional knockout models (Lee et al., 2016, 2014).

Clarifications required:

• Were different males, but the same females, used for acute and repeated exposure groups? If so, please justify. If not, please clarify. The same males and females were used for the acute and repeated exposure groups. This is now clarified in the text (pg. 6).

• Why was the 1 g/kg ethanol dose chosen? We performed a dose-response examining changes in network activity in response to four different doses of alcohol (0.5, 1.0, 1.5, 2.0 g/kg i.p.). These experiments determined that that a 1.0 g/kg dose was capable of significantly altering the BLA network state without producing lethargy or sedation in the mice. Therefore, we chose this concentration for our experiments throughout this study. This is now stated in the text (pg. 8).

• When were tissue samples collected for IHC in comparison to the last dose of alcohol? What were BECs at this time point? Why was this time point chosen? Tissue samples were collected 30 minutes after the last dose of alcohol as that is the amount of time, we observe BLA LFP effects after alcohol administration. We collected BECs at 15 minutes after administration because we wanted to show the BEC levels at the midpoint of the effect on BLA LFPs. This information is now provided in the methods section (pg. 6).

• The authors mention the role of ovarian hormones in drinking, mood, oscillatory state and expression of δ-GABAARs, but do not mention the cycle of the mice used in these experiments. Female mice in our care are maintained in an acyclic state without exposure to males. This is now stated in the manuscript (pg. 4). Therefore, we did not examine the impact of the estrous cycle on the outcome measures in the current study, although we agree that this is an interesting avenue of research for future studies.

• What is the arrow in Figure 1A pointing at? The arrow in the original version of Figure 1 was indicating the placement of the LFP electrode. We have removed the arrow from the new version of this figure (Figure 1).

• Figure 1 and 2 should clearly indicate the use of male (Fig 1) and female (Fig 2) mice. In addition, Figure 2 should include a power spectrum curve as shown in Figure 1 and a dotted line should be added to Fig 2B. We added a power spectrum curve for both male and female C57BL/6J mice (Figure 1-1) and clearly indicated on all figure panels the sex and genotype of the mice being represented.

• Figure 1C, text results do not match the figure. High theta is reduced, but low/high gamma are increased. We thank the reviewer for pointing out this error in the manuscript. This has been corrected in the revised manuscript text (pg. 8).

• In Figure 2D, an ethanol effect is reported, but it looks as if reduction in power area was occurring prior to ethanol treatment, and it reached asymptote with alcohol. Please address/clarify. The Reviewer is correct that there is a pronounced and reproducible effect of the vehicle treatment on the BLA network state, which we have previously reported (Antonoudiou et al., 2021) and attribute to the stress of the injection. Thus, the effects of the ethanol treatment are normalized to baseline and compared to the vehicle injections. The vehicle injection increases gamma frequency and alcohol blunts the increase, overall producing a suppression of power compared to the vehicle injection. This has been clarified in the text (pgs. 8 and 9).

• Line 257: How is tolerance being defined? Tolerance to which effects? The authors thank the Reviewer for pointing out issues relating to the terminology used in the text and the discussion around tolerance. We have changed our discussion of the effects of repeated alcohol administration on male BLA LFPs to sensitization rather than discussing tolerance of female mice. This study did not directly measure behavioral sensitization or any specific type of tolerance. We made an observation that the same dose of alcohol was producing a larger effect on BLA LFPs across days in males only and interpreted that as a sensitization to the effects of alcohol. We further discuss how tolerance is related to downregulation of the delta subunit, an effect we see in female C57, and suggest that is a reason why repeated alcohol does not have an effect on female BLA LFPs.

• Include the catalog number and other relevant details for antibodies This has been added to the methods (pg. 7).

• Line 196-198: Is this comparing C57 and Gabrd animals? Yes, we first describe the effect of alcohol on the BLA network state and then directly compare between genotypes. This has been clarified in the manuscript (pg. 10).

Minor issues

• PV+ cells operate in the gamma frequency range, and there were several sex differences and alcohol effects observed in this frequency range. The Discussion, however, centers on overall changes in oscillatory states of the network, while most changes occurred in the gamma frequency. We have added more discussion specifically about gamma frequency and what a reduction of power in this range may mean and the potential role of PV interneurons (pg. 18).

• Lines 251 and 270: Re-word second sentence. This has been edited in the manuscript.

• Lines 259-269: Please strengthen the rationale for examining sex differences in PV δ-GABAAR’s regulation of network oscillations. We clarified the rationale for these experiments, highlighting the evidence that alcohol exposure and sex hormones can change the expression of GABAAR subunits, including the delta subunit. Alcohol has been shown to modulate tonic inhibition via delta-GABAARs, which we have demonstrated are uniquely expressed on PV interneurons in the BLA (Antonoudiou et al., 2021). Tonic inhibition from PV GABAergic interneurons is necessary for oscillation generation and synchrony. Therefore, we were motivated to investigate potential sex mediated differences in delta expression on PV interneurons to understand if that could explain the differences, we observe in BLA LFPs. This is now clearly stated in the Introduction (pg. 4, 13, 17).

• Line 267: Remove the word “changes”. This word has been removed.

• The labeling of supplementary figures is confusing. There are no numbers on any figures and attachments do not specify figure or table number, making it difficult to link results to figures. Figures have been labeled according to eNeuro’s guidelines.

• Please proofread for typos and confusing wording. The paper has been proofread and edited by all authors.

References

Agoglia, A. E., Zhu, M., Ying, R., Sidhu, H., Natividad, L. A., Wolfe, S. A., ... Herman, M. A. (2020). Corticotropin-Releasing Factor Receptor-1 Neurons in the Lateral Amygdala Display Selective Sensitivity to Acute and Chronic Ethanol Exposure. ENeuro, 7(2), 1-18. https://doi.org/10.1523/ENEURO.0420-19.2020

Antonoudiou, P., Colmers, P. L. W., Walton, N. L., Weiss, G. L., Smith, A. C., Nguyen, D. P., ... Maguire, J. L. (2021). Allopregnanolone Mediates Affective Switching Through Modulation of Oscillatory States in the Basolateral Amygdala. Biological Psychiatry. https://doi.org/10.1016/j.biopsych.2021.07.017

Lee, V., MacKenzie, G., Hooper, A., & Maguire, J. (2016). Reduced tonic inhibition in the dentate gyrus contributes to chronic stress-induced impairments in learning and memory. Hippocampus, 26(10), 1276-1290. https://doi.org/10.1002/HIPO.22604

Lee, V., Sarkar, J., & Maguire, J. (2014). Loss of Gabrd in CRH neurons blunts the corticosterone response to stress and diminishes stress-related behaviors. Psychoneuroendocrinology, 41, 75-88. https://doi.org/10.1016/j.psyneuen.2013.12.011

Sarkar, J., Wakefield, S., Mackenzie, G., Moss, S. J., & Maguire, J. (2011). Neurosteroidogenesis Is Required for the Physiological Response to Stress: Role of Neurosteroid-Sensitive GABA A Receptors. The Journal of Neuroscience, 31(50), 18198-18210. https://doi.org/10.1523/JNEUROSCI.2560-11.2011
