# Supplementary figures and images for: Sex Differences in the Alcohol-Mediated Modulation of BLA Network States
Source: eNeuro. 2022 Jul 7;9(4):ENEURO.0010-22.2022. doi: 10.1523/ENEURO.0010-22.2022 (PMC9275151; doi:10.1523/ENEURO.0010-22.2022)

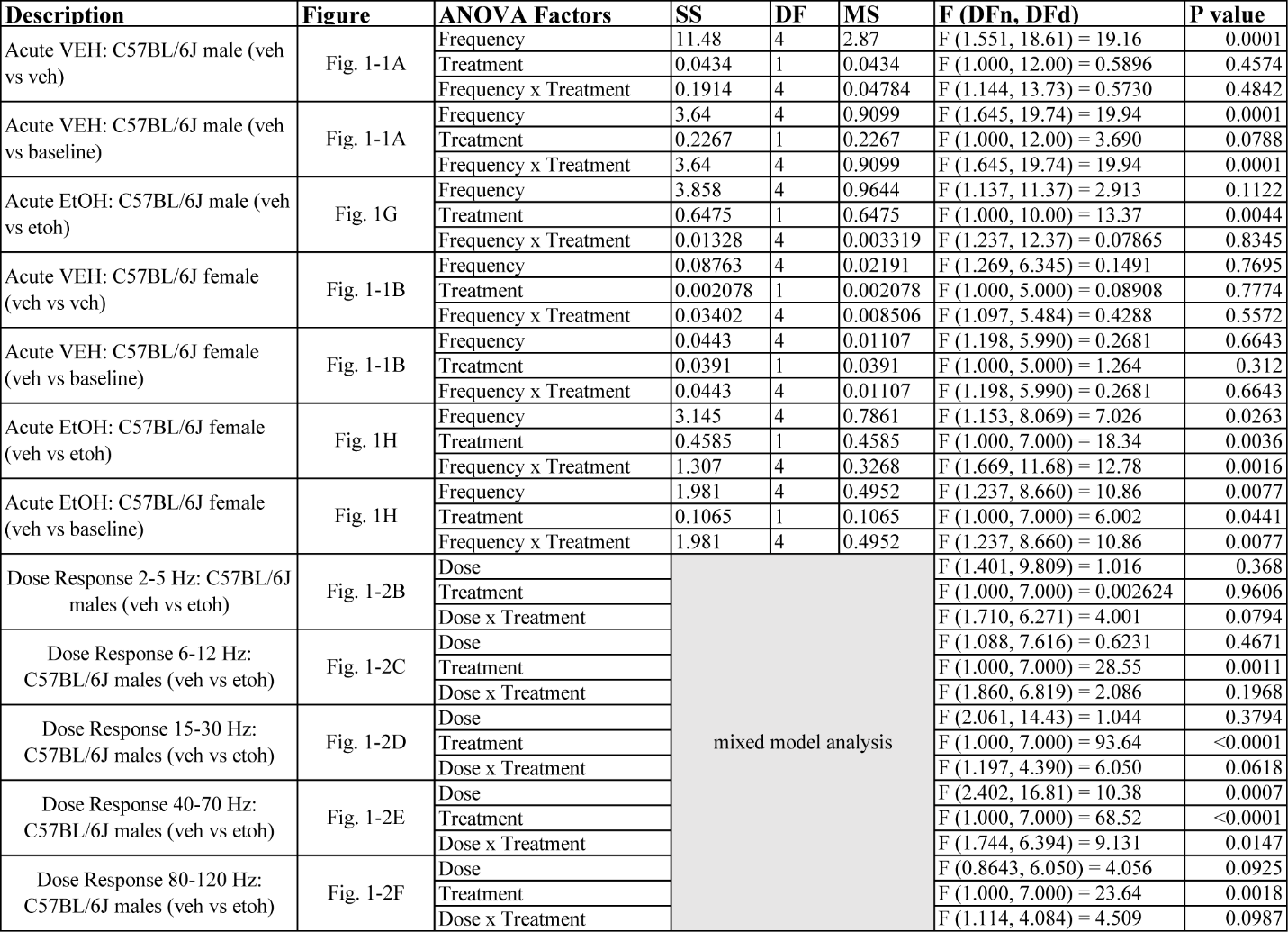

Supplement: Table 1-1 — Summary of ANOVAs for acute alcohol experiments in C57BL/6J mice. Download Table 1-1, DOCX file. [file enu-eN-NWR-0010-22-s03.docx]

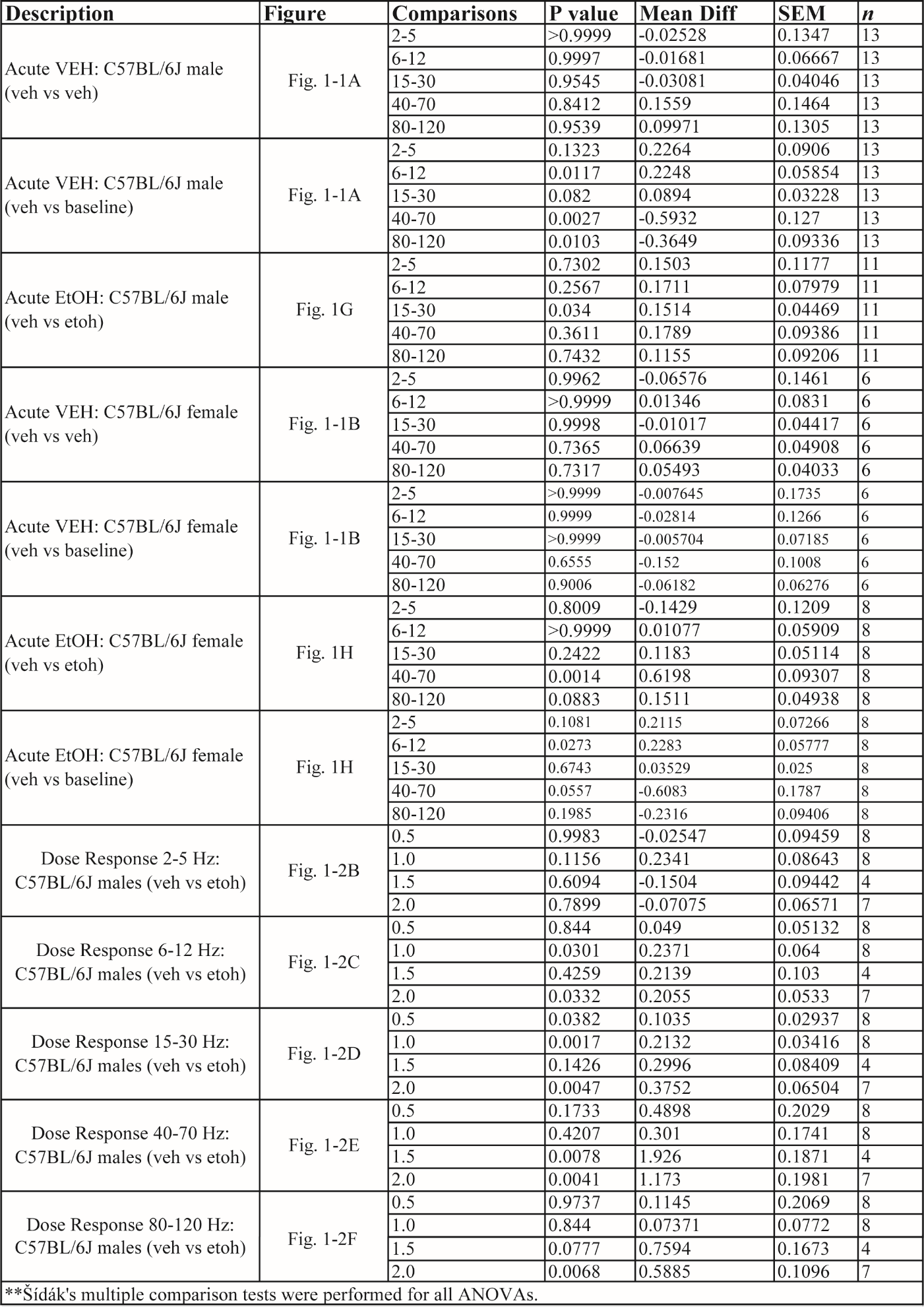

Supplement: Table 1-2 — Summary of multiple-comparison tests for acute alcohol experiments in C57BL/6J mice. Download Table 1-2, DOCX file. [file enu-eN-NWR-0010-22-s04.docx]

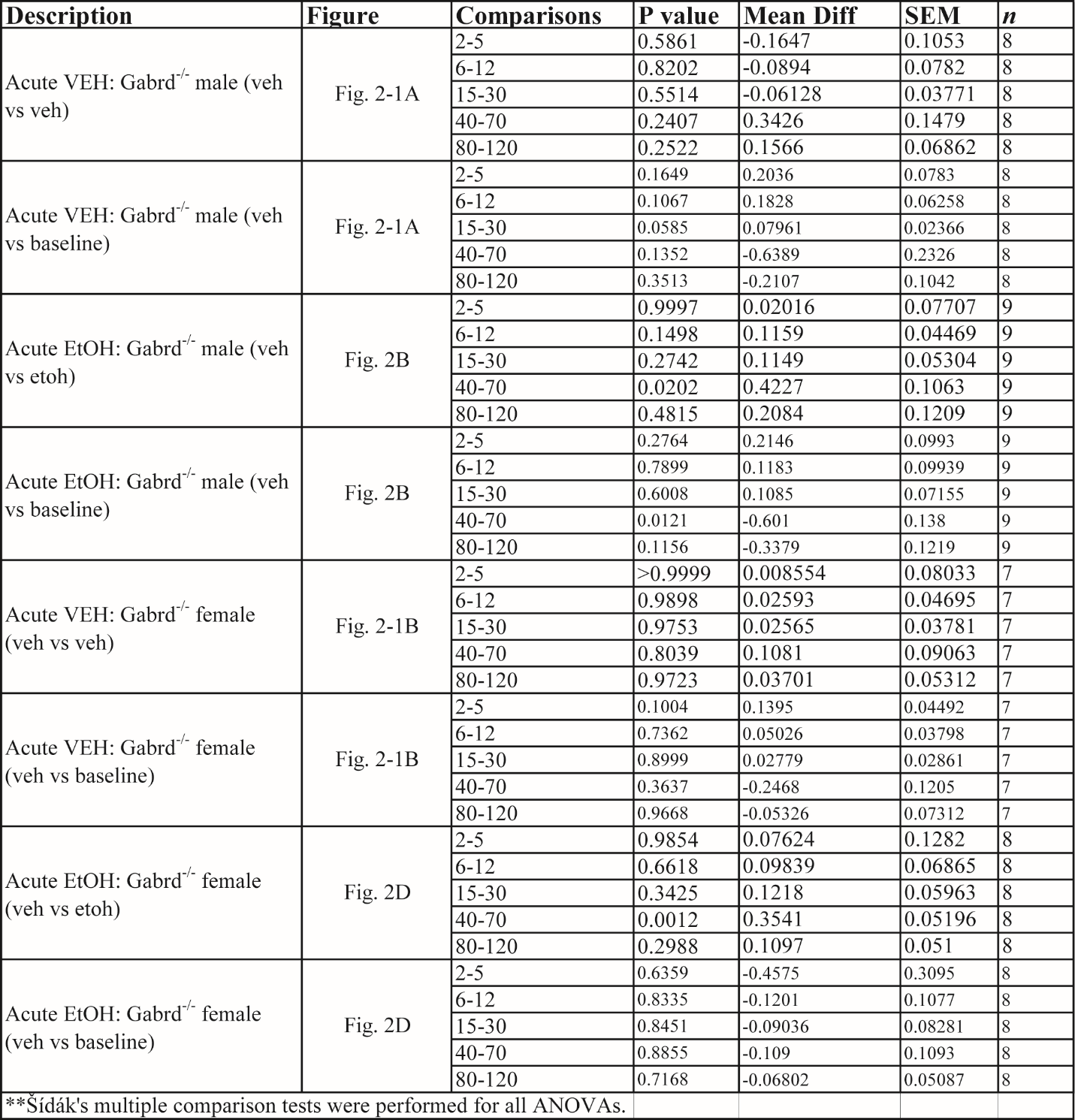

Supplement: Table 2-2 — Summary of multiple-comparison tests for acute alcohol experiments in Gabrd– /– mice. Download Table 2-2, DOCX file. [file enu-eN-NWR-0010-22-s07.docx]

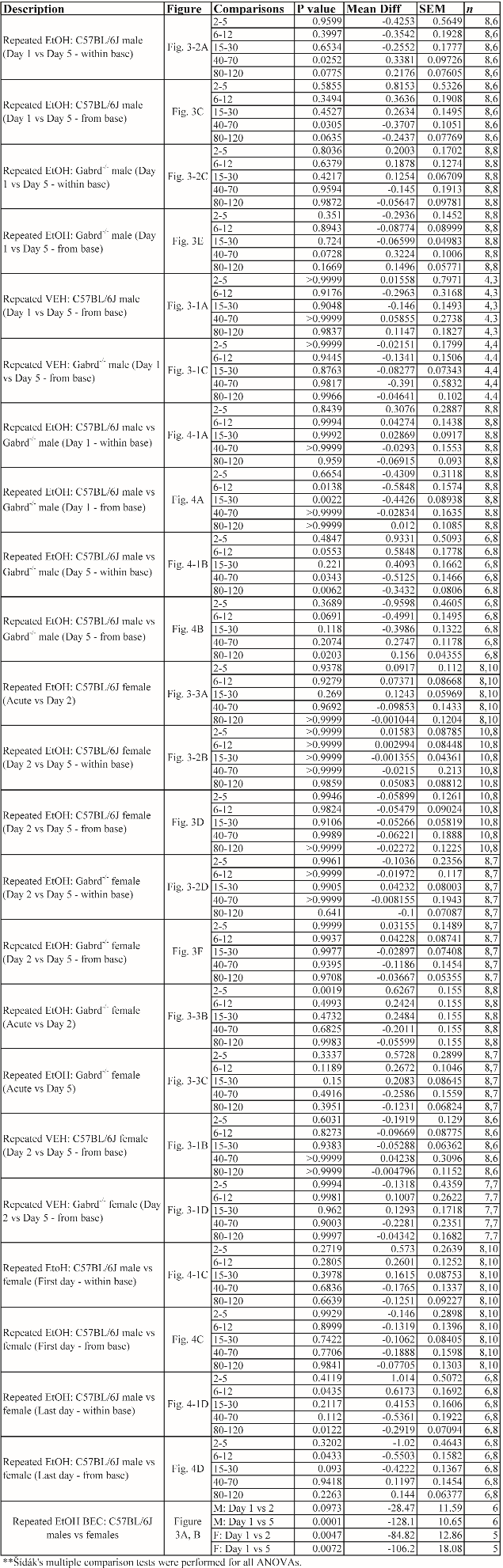

Supplement: Table 3-2 — Summary of multiple-comparison tests for repeated alcohol experiments. Download Table 3-2, DOCX file. [file enu-eN-NWR-0010-22-s12.docx]

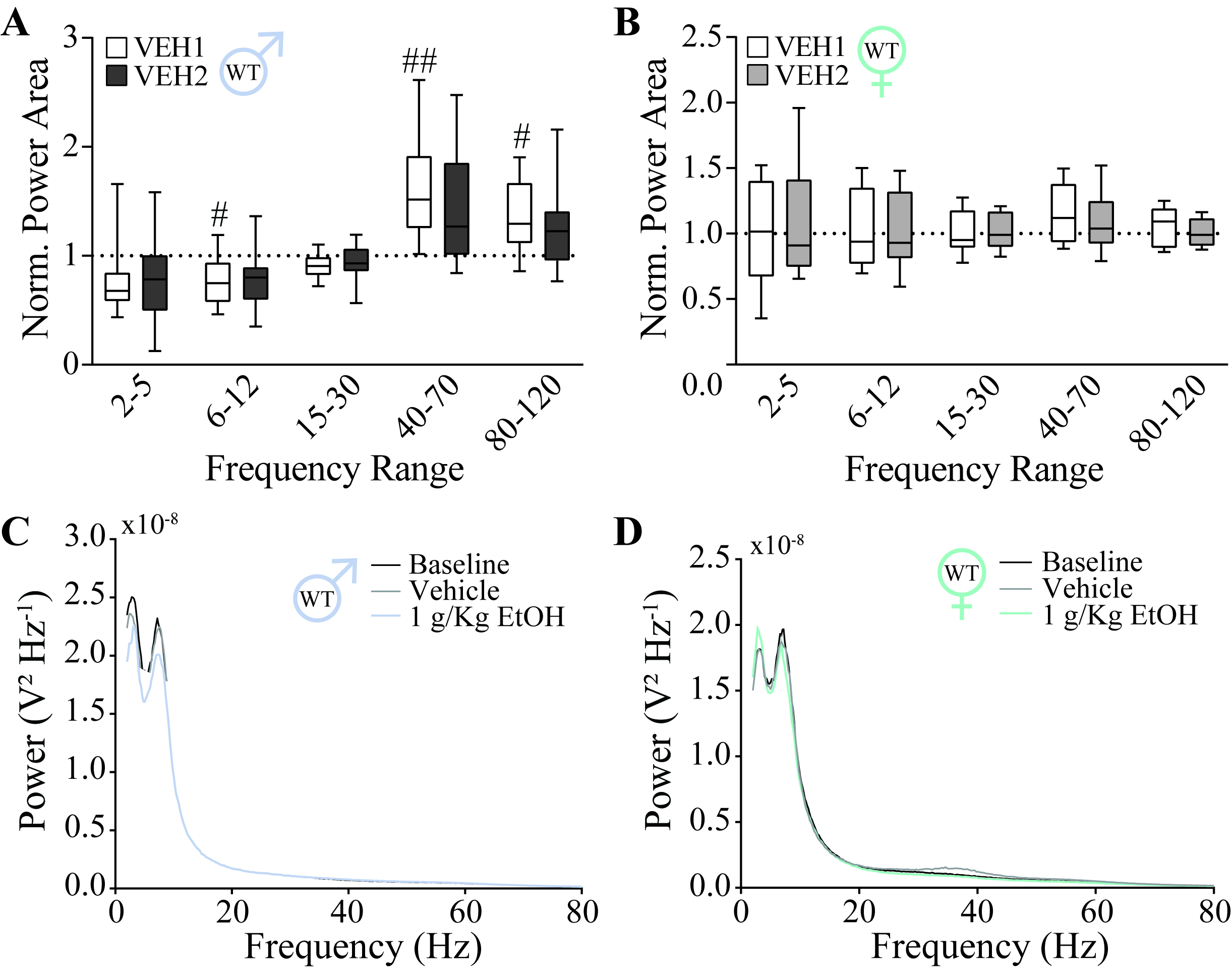

Supplement: Figure 1-1 — Acute vehicle exposure does not alter BLA network activity in C57BL/6J mice. A, B, Normalized power area for vehicle/vehicle acute exposure in male (n = 13; A) and female (n = 6; B) mice. C, D, Power spectral density of baseline, vehicle, and 1 g/kg alcohol injection over 0–80 Hz in male mice (C) and female mice (D). #p < 0.05, ##p < 0.01 versus baseline. Download Figure 1-1, TIF file. [file enu-eN-NWR-0010-22-s01.tif]

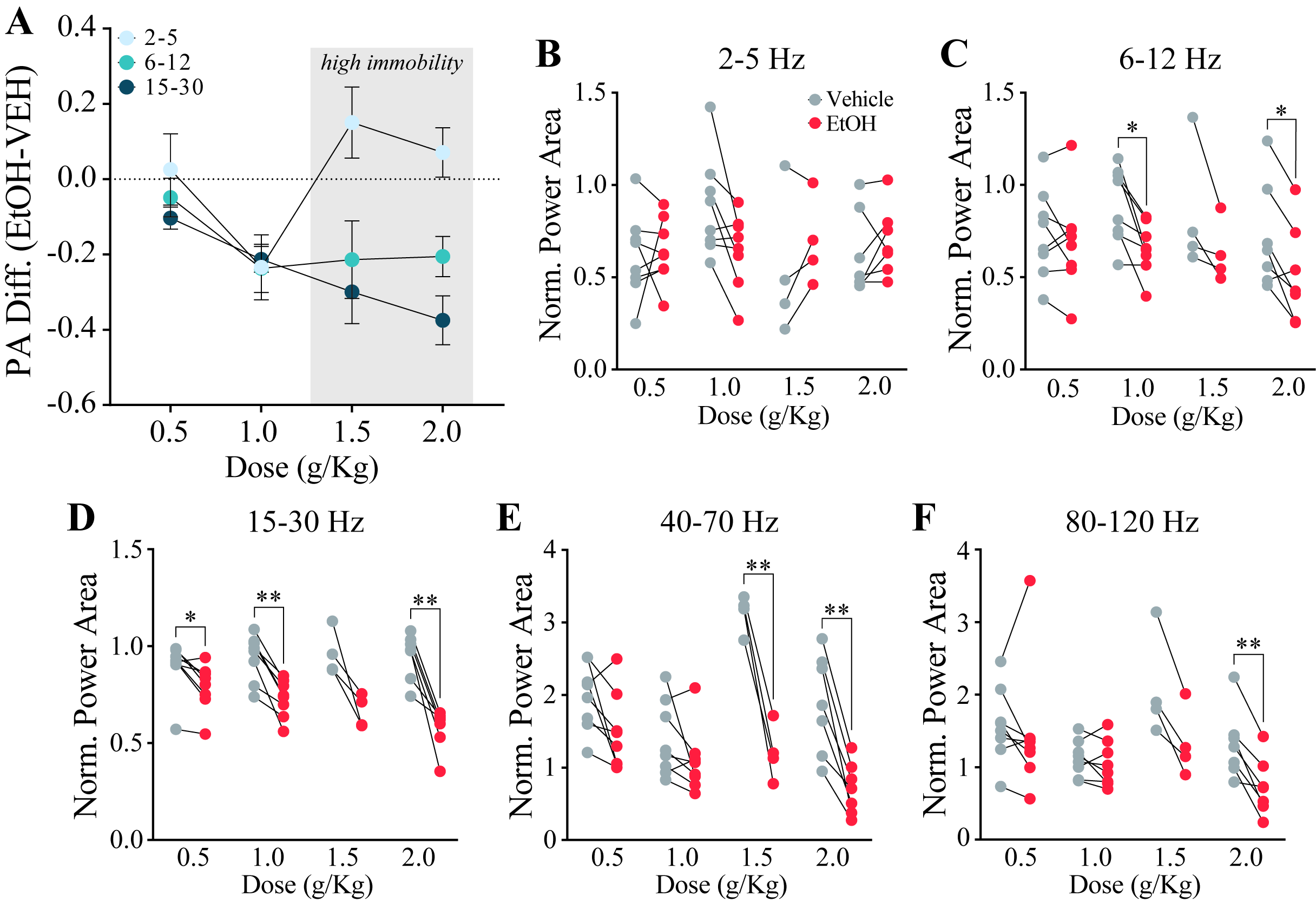

Supplement: Figure 1-2 — Effects of an alcohol dose response on BLA network states. A, Power area difference of 2–5, 6–12, and 15–30 Hz frequency ranges across alcohol doses in male C57BL/6J mice (0.5, n = 8; 1.0, n = 8; 1.5, n = 4; 2.0, n = 7). B–F, Shaded region indicates doses that caused high immobility in mice. Normalized power area for vehicle/alcohol exposure across doses for 2–5 Hz (B), 6–12 Hz (C), 15–30 Hz (D), 40–70 Hz (E), and 80–120 Hz (F). *p < 0.05, **p < 0.01 versus vehicle. Download Figure 1-2, TIF file. [file enu-eN-NWR-0010-22-s02.tif]

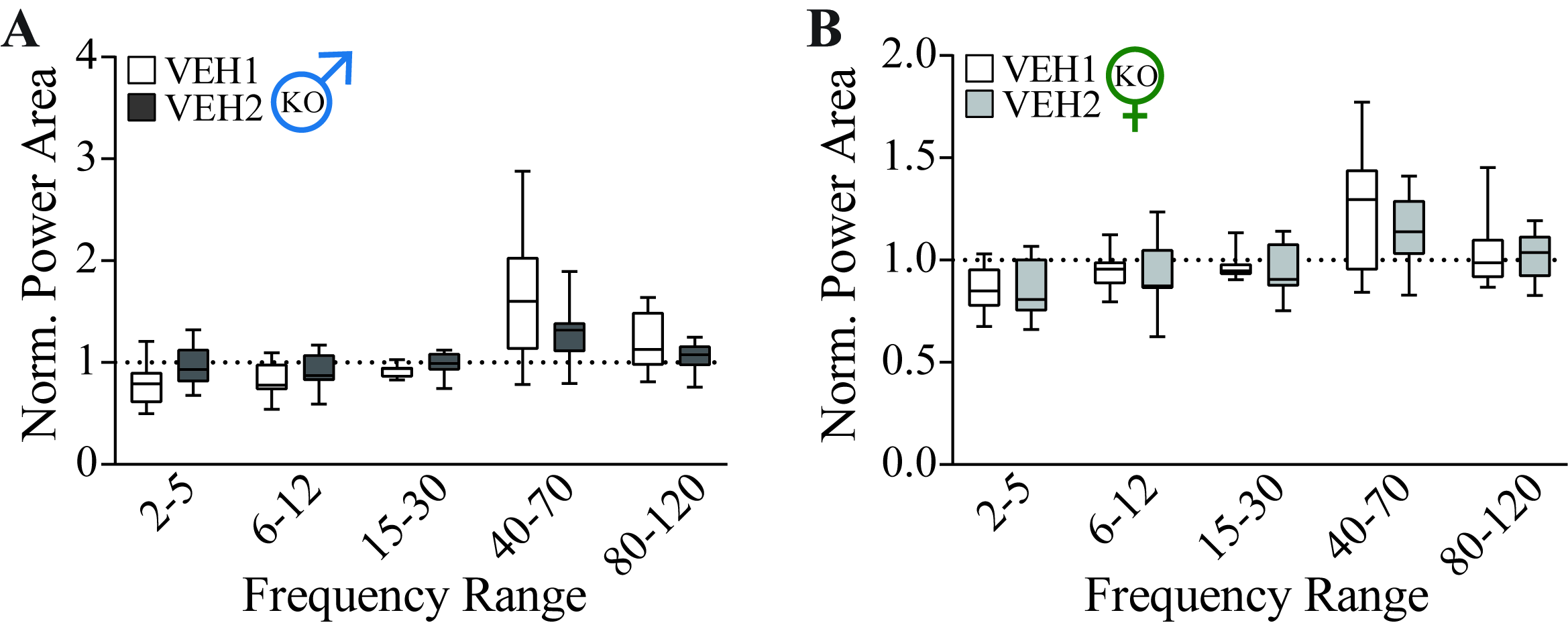

Supplement: Figure 2-1 — Acute vehicle exposure does not alter BLA network activity in Gabrd– /– mice. A, B, Normalized power area for vehicle/vehicle exposure in male mice (n = 8; A) and female mice (n = 7; B). Download Figure 2-1, TIF file. [file enu-eN-NWR-0010-22-s05.tif]

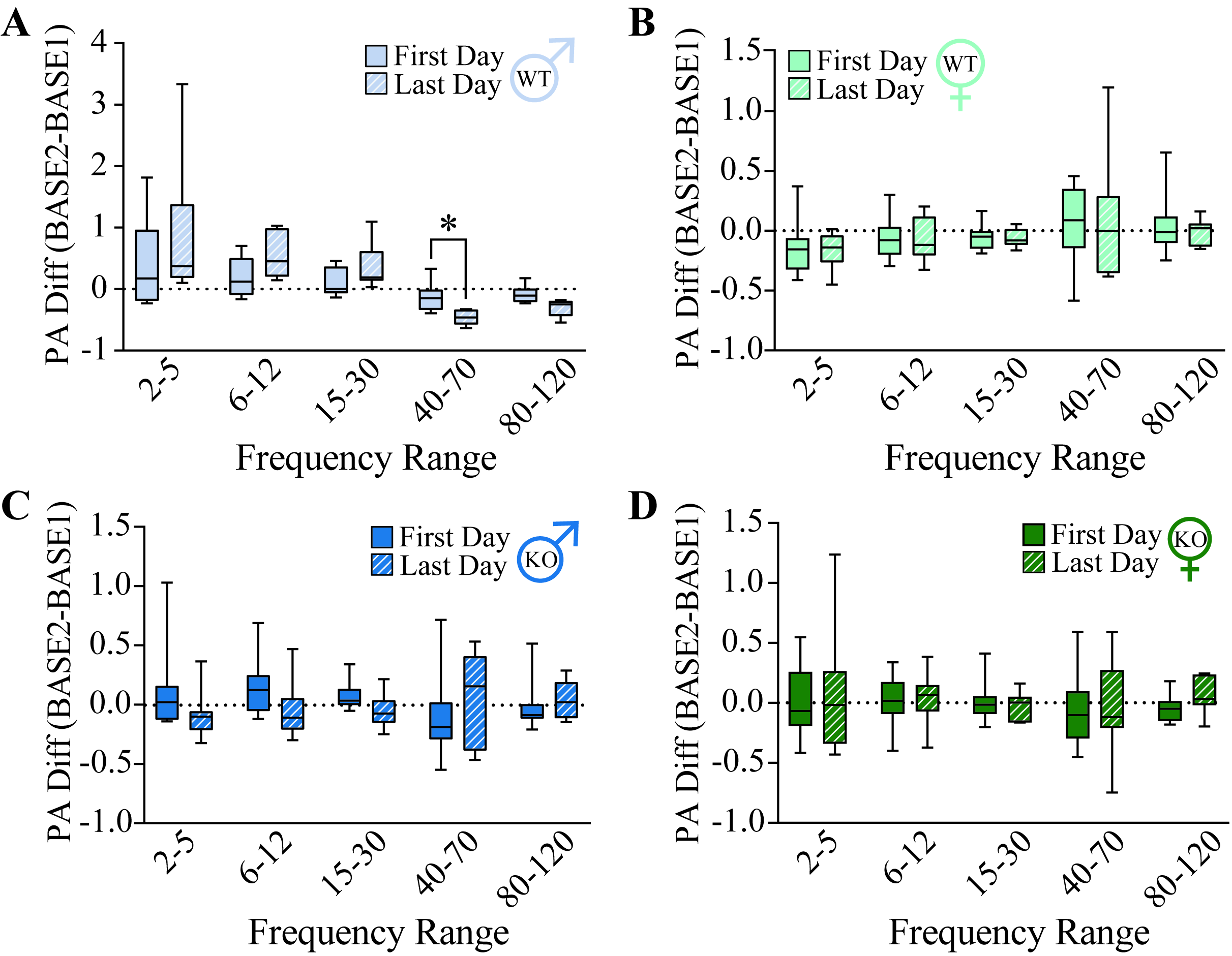

Supplement: Figure 3-2 — Repeated alcohol exposure modulates baseline network activity in male C57BL/6J mice. A, B, Change in baseline on the first and last day of alcohol exposure in male (A) and female (B) C57BL/6J mice and male (C) and female (D) Gabrd– /– mice. *p < 0.05. Download Figure 3-2, TIF file. [file enu-eN-NWR-0010-22-s09.tif]

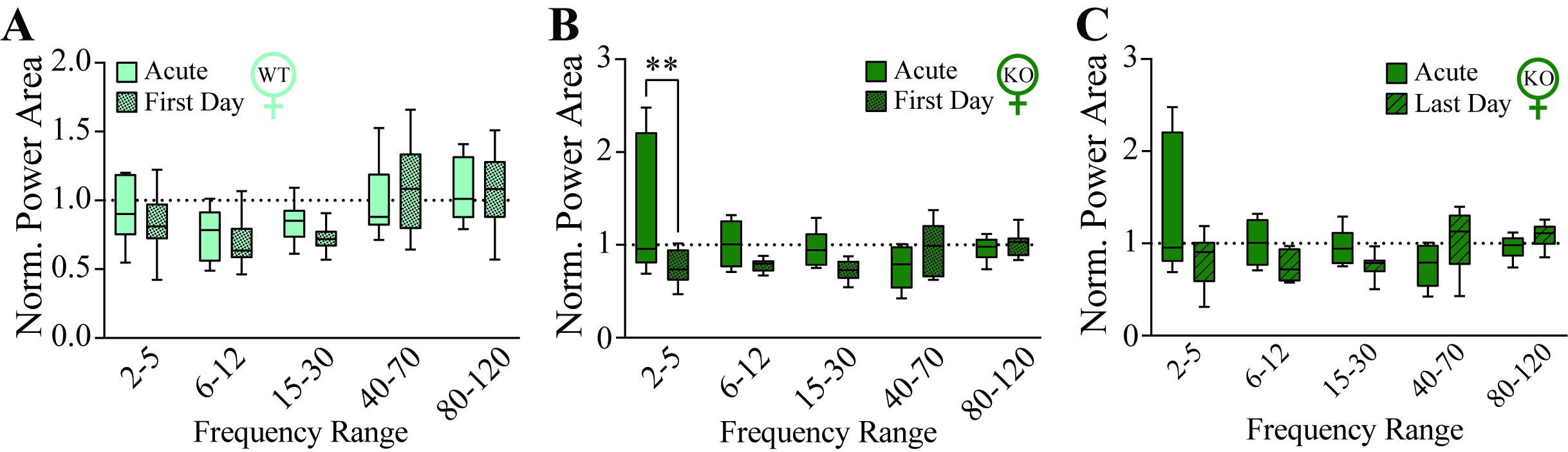

Supplement: Figure 3-3 — Effects of acute alcohol exposure on day 1 (acute) and day 5 do not differ in female Gabrd– /– mice. A, Normalized power area of alcohol injections during the acute alcohol injection and day 2 in female C57BL/6J mice (n = 10). B, Normalized power area of alcohol injections during the acute alcohol injection and day 2 in female Gabrd– /– mice. B, Normalized power area of alcohol injections during acute alcohol injection and day 5 in female Gabrd– /– mice. **p < 0.01. Download Figure 3-3, TIF file. [file enu-eN-NWR-0010-22-s10.tif]

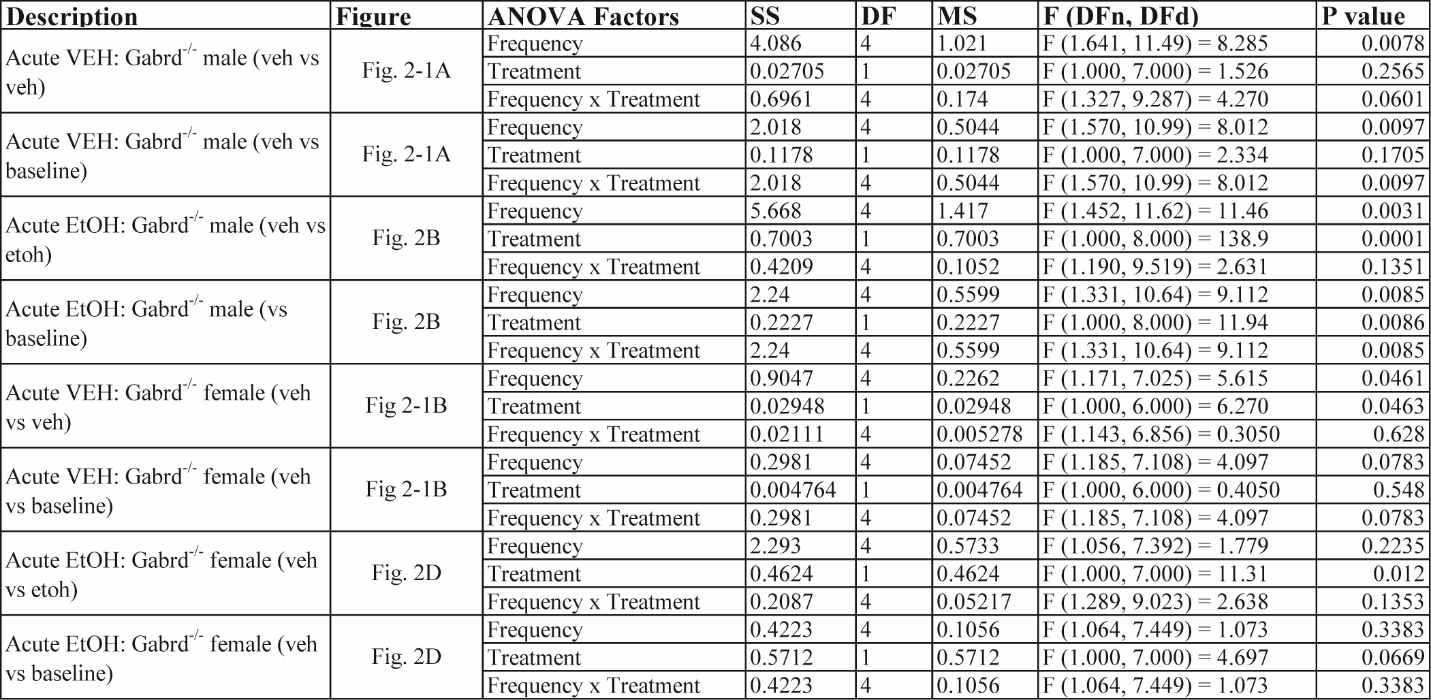

Supplement: Table 2-1 — Summary of ANOVAs for acute alcohol experiments in Gabrd– /– mice. Download Table 2-1, DOCX file. [file enu-eN-NWR-0010-22-s06.docx]

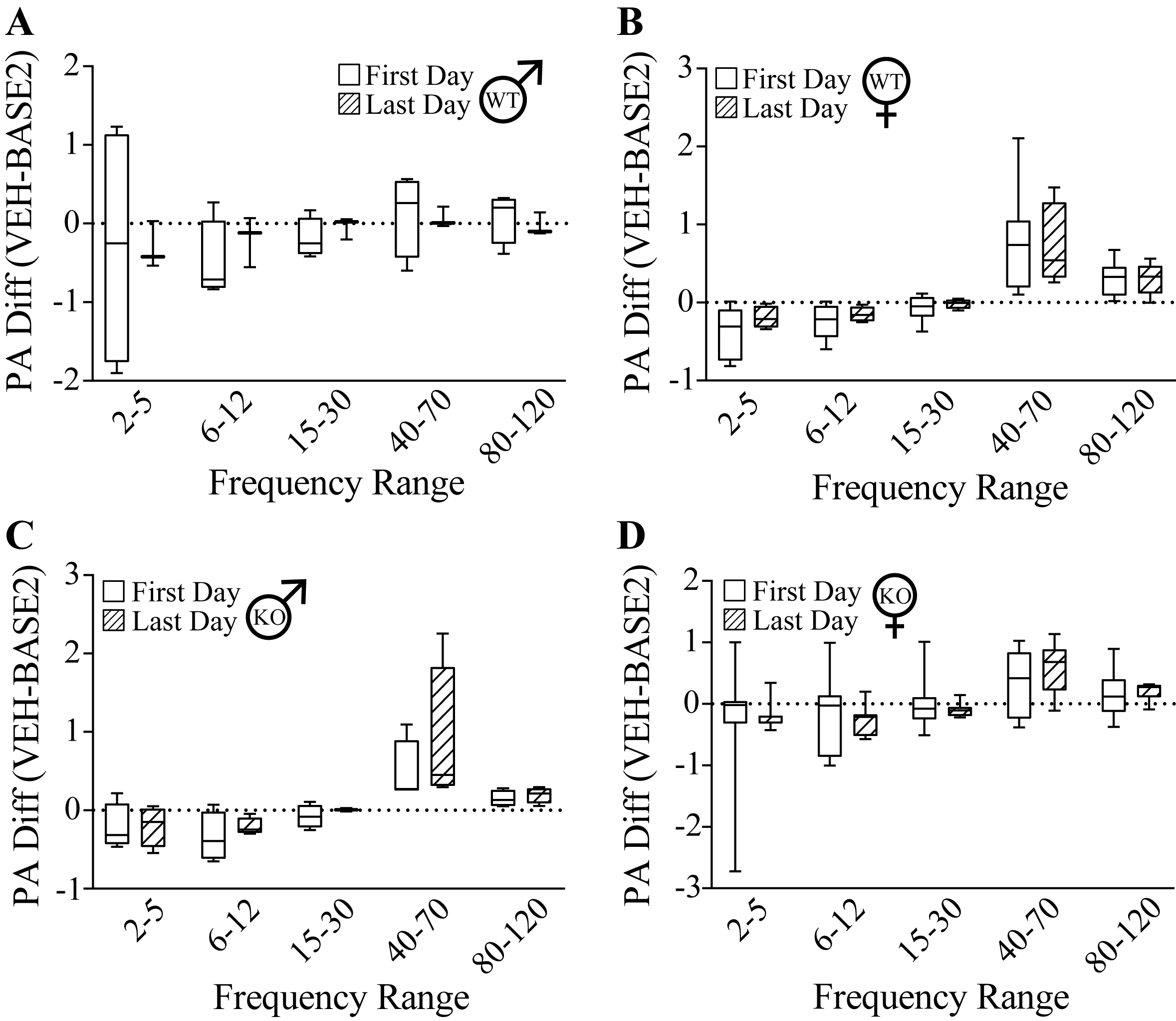

Supplement: Figure 3-1 — Repeated vehicle exposure does not change BLA network activity in C57BL6/J and Gabrd– /– mice. A–D, Power area difference between vehicle and baseline for the first and last days of exposure in male (first day, n = 4; last day, n = 3; A) and female (first day, n = 5; last day, n = 6; B) C57BL/6J mice and male (first day, n = 4; last day, n = 4; C) and female (first day, n = 7; last day, n = 7; D) Gabrd– /– mice. Download Figure 3-1, TIF file. [file enu-eN-NWR-0010-22-s08.tif]

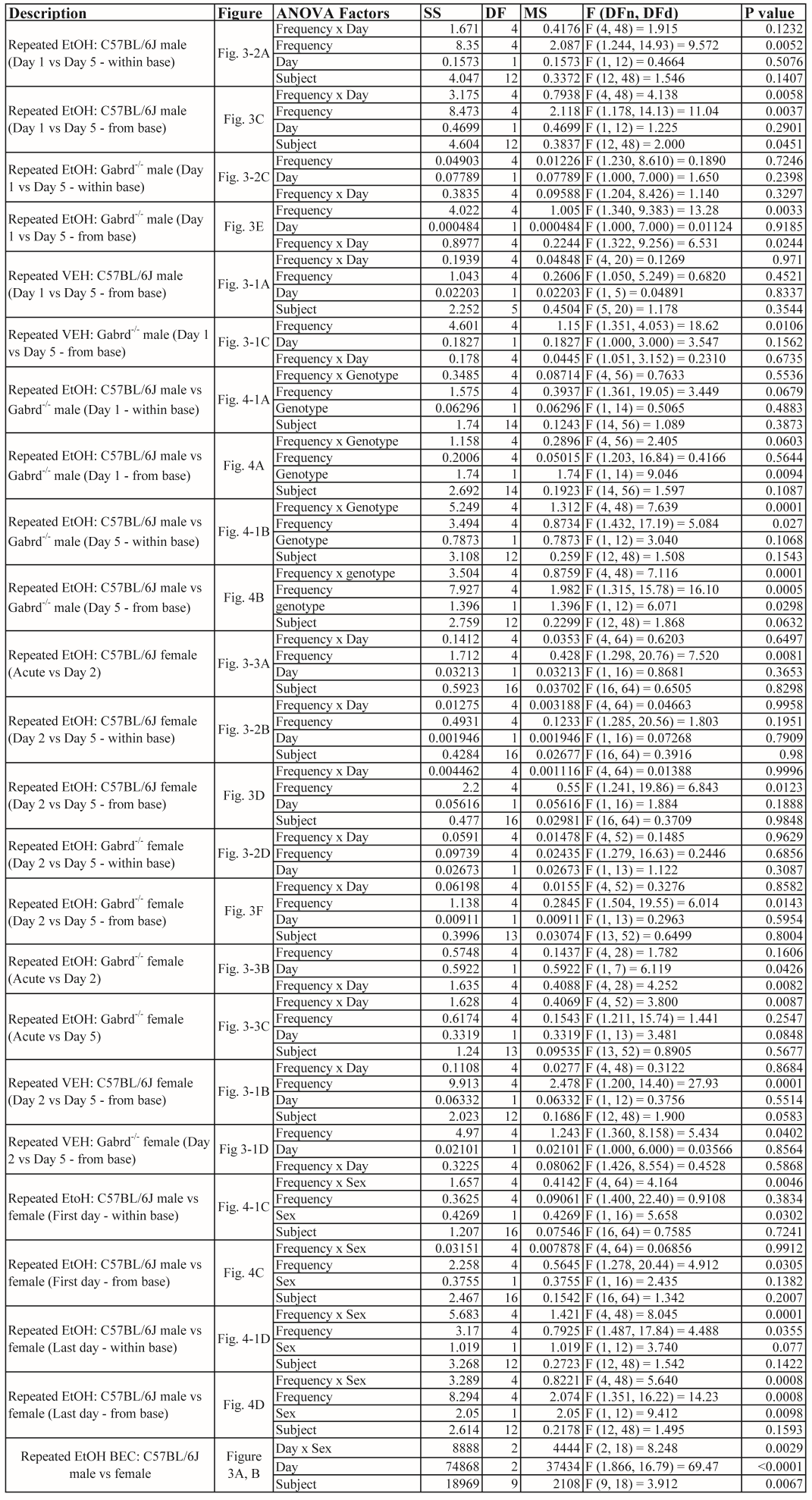

Supplement: Table 3-1 — Summary of ANOVAs for repeated alcohol experiments. Download Table 3-1, DOCX file. [file enu-eN-NWR-0010-22-s11.docx]
